# Synthesis and applications of theranostic oligonucleotides carrying multiple fluorine atoms

**DOI:** 10.7150/thno.37936

**Published:** 2020-01-01

**Authors:** Valeriy G. Metelev, Alexei A. Bogdanov

**Affiliations:** 1 Laboratory of Molecular Imaging Probes, Department of Radiology, University of Massachusetts Medical School, Worcester MA, USA.; 2 Department of Chemistry, Moscow State University, Moscow, Russian Federation.; 3 Laboratory of Molecular Imaging, A.N. Bakh Institute of Biochemistry, Federal Research Center "Fundamentals of Biotechnology" of the Russian Academy of Sciences, Moscow.; 4 Department of Bioengineering and Bioinformatics, Moscow State University, Moscow.

**Keywords:** oligonucleotide, perfluorinated, drug delivery, sensor, fluorescence, magnetic resonance spectroscopy

## Abstract

The use of various oligonucleotide (ON) syntheses and post-synthetic strategies for targeted chemical modification enables improving their efficacy as potent modulators of gene expression levels in eukaryotic cells. However, the search still continues for new approaches designed for increasing internalization, lysosomal escape, and tissue specific delivery of ON. In this review we emphasized all aspects related to the synthesis and properties of ON derivatives carrying multifluorinated (MF) groups. These MF groups have unique physico-chemical properties because of their simultaneous hydrophobicity and lipophobicity. Such unusual combination of properties results in the overall modification of ON mode of interaction with the cells and making multi-fluorination highly relevant to the goal of improving potency of ON as components of new therapies. The accumulated evidence so far is pointing to high potential of ON probes, RNAi components and ON imaging beacons carrying single or multiple MF groups for improving the stability, specificity of interaction with biological targets and delivery of ONs *in vitro* and potentially *in vivo*.

## Introduction

Chemical modification of oligonucleotides (ON) is universally recognized as the most facile strategy for improving their ability to alter the levels of gene expression *in vitro* and *in vivo* 1. In this regard, unique properties of fluorine continue to attract attention of multiple research groups which investigated novel synthetic materials for biomedical applications 2. In the past the introduction of 2'-fluorine atoms into the composition of ON during the synthesis was successfully used for improving stability and kinetic properties of synthetic oligoribonucleotide-based ribozymes 3. Further increase of fluorine incorporation into the backbone of ON analogs was also considered. Modification of the backbone of peptide nucleic acids (PNA) with only two fluorines per three nucleobases has been shown to change the properties of the resultant PNA so dramatically that their ability to cross cellular membranes showed a marked improvement 4. Subsequently, multifluorinated (MF) groups that have an advantage of high local density of fluorine atoms were also investigated as potential ON modifiers. Unlike more traditional 2'- fluorination of nucleosides MF were relatively recently introduced as components of new materials based on chemically modified nucleic acids 5-7. Such chemical modification of nucleic acids does not require extensive substitution of all backbone components and usually includes a single point chemical modification 8. The resultant multifluorinated oligonucleotides (MF-ON) embody a combination of two very different essential parts: 1) multifluorinated component(s), which are simultaneously highly hydrophobic and lipophobic (repelling hydrocarbons) giving MF-ON a tendency to engage in self-assembly into various supramolecular structures; 2) relatively hydrophilic and water- soluble component (i.e. ON or ON analog) responsible for specific interactions with target nucleic acids or proteins. In general, synthetic strategies and microenvironment play crucial roles and in multiple aspects they guide the assembly of oligonucleotide components in various supramolecular structures 9. The properties of these structures as well as MF-ON building blocks can potentially assist in designing oligonucleotide conjugates with diverse architecture, fluorine-based NMR spectroscopic/imaging tags and improved delivery *in vitro* and *in vivo* resulting in an enhanced therapeutic potential 8. In this review we set forth to address various aspects of synthesis and properties of MF-ON modified with fluorocarbon-containing (perfluoroalkyl) moieties such as covalently linked tags and multiple “tails” since the goal of maximizing the local density of fluorine atoms within the molecule is important for intracellular delivery and NMR spectroscopy due to specific fluorine nuclear spin relaxation properties.

## Synthesis of ON containing MF moieties

The great majority of fluoro-modified oligonucleotides reported so far were obtained by using chemical synthesis including: a) standard solid phase ON synthesis requiring the use of specialty synthons for introducing perfluoroalkyl moieties; b) post-synthetic conjugation of fluorocarbon-containing groups by using premade oligonucleotides containing suitable reactive or activatable moieties.

### Incorporation of MF groups in MF-ON composition during synthesis

In the majority of cases MF groups were included into ON composition as ON termini modifiers. Those reactions involve the use synthons or commercially available multifluorine-containing reagents for standard solid phase synthesis. Several examples of various MF-synthons were suggested so far, which are combined in Table 1 of this review for convenience. For example, to study intracellular transport of modified ONs a commercially available synthon **Ia** was used for the synthesis of a 21-base long phosphorothioate-bond stabilized ONs 5'-derivatives. The resultant MF-ON contained perfluorooctylpropyl (CF_3_(CF_2_)_7_(CH_2_)_3_) residues as well as aminoethyldiethylene glycol linkers at various internucleoside positions 10(see structure MF-ON **1a**, Table 2). Amino linkers were used for conjugating various organic fluorophores that enables sensitive detection of MF-ON distribution within the cells *in vitro*.

The synthesis of various fluoro-modified phosphoroamidites is relatively straightforward and can be accomplished by reacting 2-cyanoethyl chlorodiisopropylphosphoramidite with corresponding alcohols provided the latter are commercially available. The examples include 2-cyanoethyl (1H,1H,2H,2H-perlfuorodecyl)diisopropylphosphoramidite (Table 1, synthon **Ib**) 11 or 2-cyanoethyl (1H,1H,2H,2H-perlfuorooctyl)diisopropylphosphoramidite (**Ic**) that were used in the past for the synthesis of the following end-modified MF-ONs 12:

C_6_F_13_CH_2_CH_2_pATCCTTATCAAT (shown in Table 2, MF-ON** 2**) C_8_F_17_CH_2_CH_2_[p(CH_2_CH_2_O)_6_]_3_pTGCAGATAGATAGCAG, C_8_F_17_CH_2_CH_2_[p(CH_2_CH_2_O)_6_]_3_pCATCATGAATTCCATAAGCTTCATGGATCCAT (Table 2, MF-ON **3**).

Nucleoside phosphoramidites with *tert*-butyl-phenyl-1*H*,1*H*,2*H*,2*H*-perfluorodecyloxysilyl groups (synthon **II**, Table 1) were previously used to synthesize fluorous-tagged oligonucleotides, as 5-mer, 10-mer, 13-mer, 17-mer, and 19-mer long MF-ONs 13. This synthon enabled synthesis of oligonucleotides that could be easily separated from failure sequences and reportedly resulted in high recovery of the end products. To improve efficiency of MF-ON isolation from reaction mixtures 5'-O-[4,4'-dimethoxy-4''-(1H,1H,2H,2H-perfluorodecyl)trityl] group was proposed as an end-modifier instead of a standard DMTr-group. For the synthesis of 30-100-mer oligonucleotides (please see the structure of 30-mer MF-ON, Table 2, MF-ON** 4**) specially designed synthons were used (e.g. synthon **III**, Table 1) 14. This universal approach was suggested as a strategy for isolating the most or all of the full-length sequences with resultant failure sequence-free ONs.

**Scheme 1:**
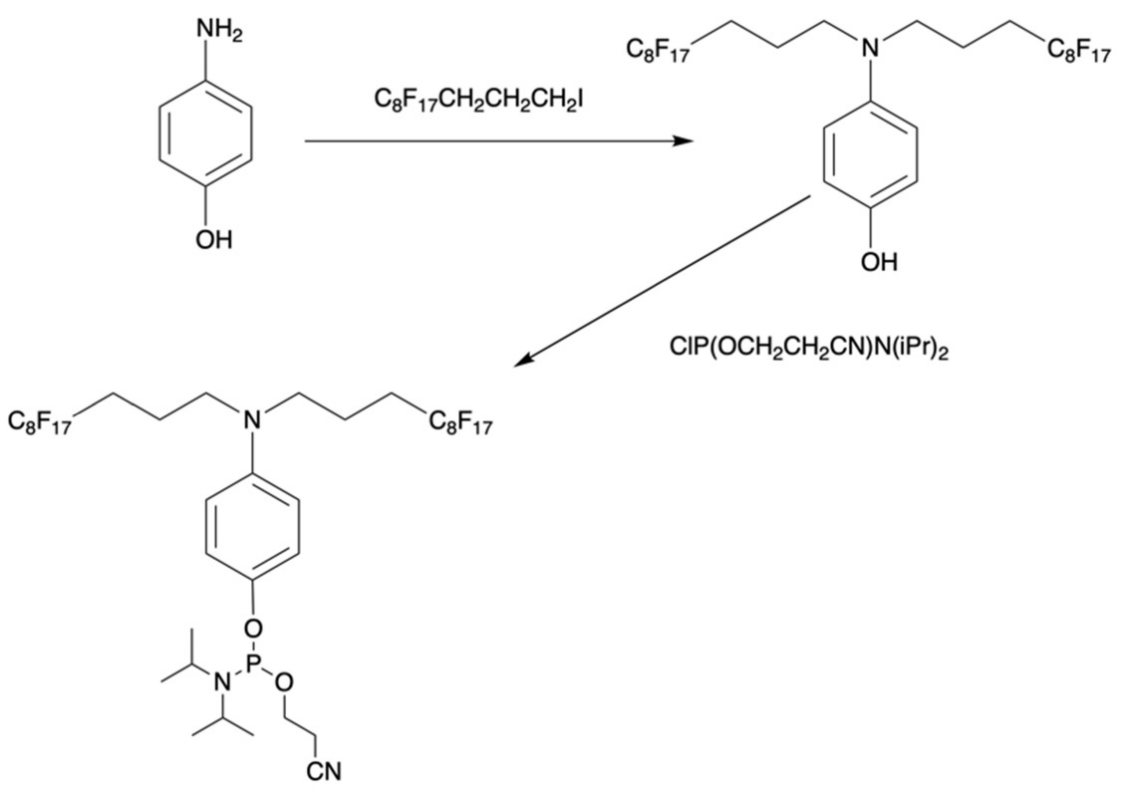
Synthesis of synthon **IV** from 4-aminophenol and heptadecafluoroundecyl iodide with further O-phosphitylation of the corresponding alkylation product.

Furthermore, perfluoroalkyl groups could be separated from ON termini by various spacers that were either hydrophilic, or hydrophobic. Importantly, the end products (i.e. MF-ON with fluorinated-groups at the 5'- terminus) can be easily separated from other components of reaction mixture due to the properties of linked MF group that results in very large differences in elution times. For example, ON carrying a single heptadecafluoroundecylcarbamoylnonyl group is eluted with a delay of 4-5 min compared to a standard DMTr-O-protected ON with identical nucleotide sequence on a standard C18 reversed phase HPLC column due to unique interactions between MF residues that potentially result in non-covalent supramolecular associates of fluorinated ON 10. Other synthons applied in the past for MF-modified ON synthesis (Table 1, **IV, V**) and especially those containing branched structures, require more elaborate and time-consuming syntheses. For example, the synthesis of synthon **IV** (Table 1) was accomplished 15 according to **Scheme 1**.

**Scheme 2:**
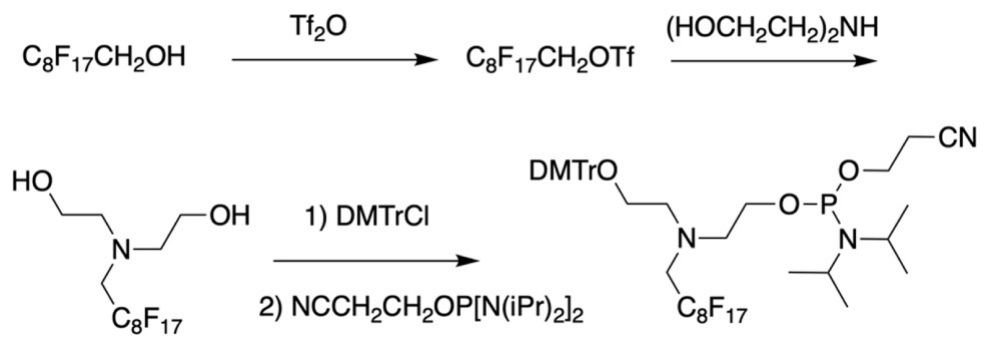
The synthesis of synthon **V** starting from converting a perfluorinated alcohol into corresponding triflate followed by heating in the presence of diethanolamine, monoprotection of the first hydroxyl group and phosphitylation of the second.

As a result by using synthon **IV** (Table 1) 15 oligonucleotides carrying pyrene as a fluorescent tag (PY) and branched MF groups such as MF-py-T_15_ (**5**), MF-TTTCCCAGCCCTC-FAM, MF-T_15_-TAMPA (Table 2, MF-ON** 5**), MF-py-T_30_, MF-T_45_ and others were obtained. Due to specifics of chemical structure, these synthons could be used either for single-step linking (**Ia-c**, **II-IV,** Table 1) of MF groups to oligonucleotides, or for multiple attachment (i.e. as multiple “tails” linked in a row, synthon **V** Table 1). The synthesis of synthon **V** (Table 1) was accomplished 16 by following **Scheme 2**.

The authors reported successful synthesis of ONs bearing MF groups at the termini as well as ON containing MF groups within the nucleotide sequence (see also MF-ON** 6a-h**, Table 2): **X**_n_TTTTTCAGTTGACCATATA, TTTTTCAGT**X**_n_TGACCATATA, TTTTTC**X**AGTTGAC**X**CATATA, TATATGGTCAACTGAAAAA**X**_n_, TATATG**X**GTCAACT**X**GAAAAA, TATATGGTCA**X**_n_ACTGAAAAA, GUCAUCACACUGAAUACCAAU**X**, where **X** is a MF, and n=1-10 in the case of ON's termini or n= 1-2 if MF groups were positioned within the sequence of the ON. The synthesis of ON with MF “segment” linked to heterocyclic moiety was accomplished by using standard solid phase oligonucleotide synthesis and synthons containing MF tert-butyl- or perfluoroalkyl- groups (examples **VI**, **VIIa-b**, Table 1).

**Scheme 3:**
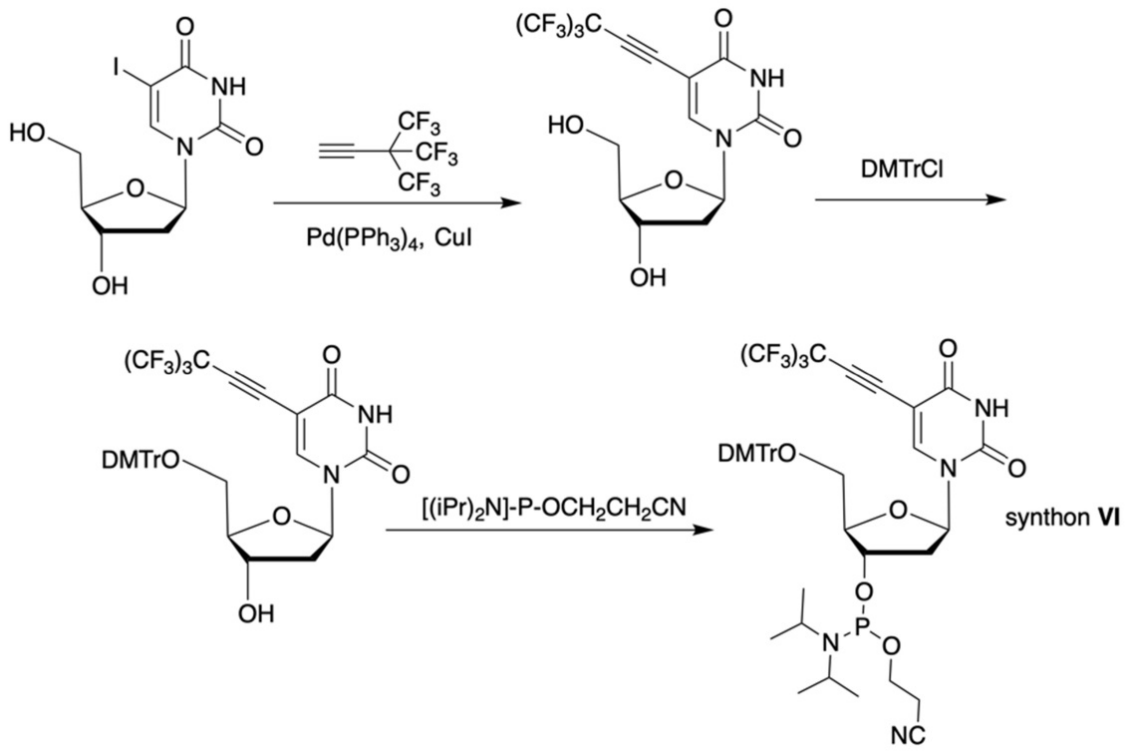
Synthesis of synthon **VI** using Sonogashira coupling reaction starting from 5-iodo-2'-deoxyuridine that included two standard synthetic steps: 1) 5'-O-tritylation, 2) 3'-O-phosphitylation.

The synthesis of synthon **VI** (Table 1) was accomplished 17 by following the** Scheme 3**.

**Scheme 4:**
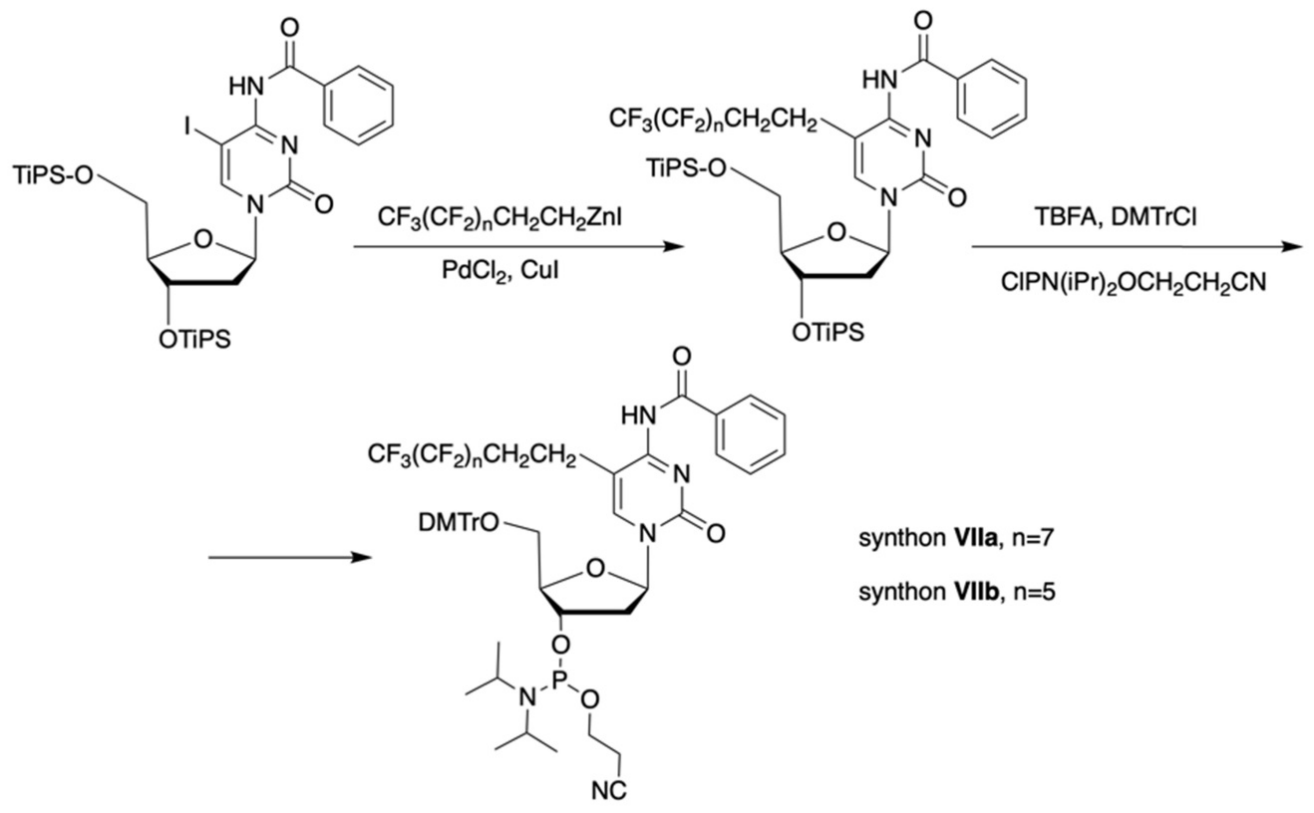
Synthesis of synthons **VIIa** and **VIIb** involving palladium-catalyzed coupling reaction between the 5-iodocytosine with organozinc reagents followed by two standard synthetic steps: 5'-O-tritylation and 3'-O-phosphitylation.

The synthon in **Scheme 3** is often considered to be the first example of a precursor specifically designed for conjugating perfluorinated tert-butyl groups to nucleic acids. In particular, oligonucleotides containing modified 2'-deoxyuridine were obtained for synthesis of NMR reporter ONs, i.e. d(CACGA*UGCGAGGTC) (Table 2, MF-ON** 7**) and d(G*UGCGCA), where *U is modified residue. The synthesis of synthons **VIIa** and **VIIb** (Table 1) was accomplished 17 by following the** Scheme 4**.

**Scheme 5:**
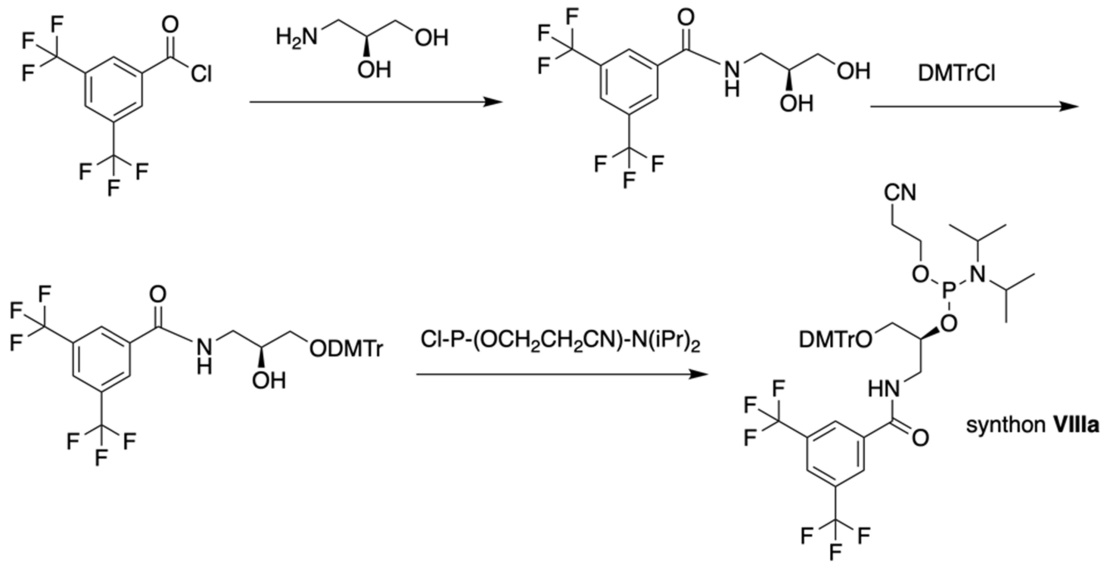
Synthesis of synthons **VIIIa** starting from acylation of (S)-3-amino-1,2-propanediol.

The separation of the modified ON products (e.g. MF-ON** 8**, Table 2) from nonfluorinated ON was feasible after incorporating MF-alkylcytosine. The incorporation of 5-C_2_F_5_ required careful elution while compounds with larger 5-C_6_F_13_ and 5-C_8_F_17_ residues could be easily separated from non-fluorous components. Structurally similar synthons **VIIIa-c** containing 3,5-bis(trifluoromethyl)phenyl groups instead of MF-alkyl moieties were used to obtain modified oligonucleotides. The synthesis of synthons **VIIIa** (Table 1) was accomplished 18 by following the **Scheme 5.**

By introducing of “**F** base” into molecular beacons the spontaneous formation of sticky stem elements was intended to yield the assembly of a “self-complementary” structure. The formation of such structure was assumed to be a result of **F**-**F** interactions of stacking flat aromatic groups. Several MF-ON types were synthesized containing multiple “**F** bases” that had a general formula of: fluorophore1-[**F**]_n_[TCTAAATCACTATGGTCGC][**F**]_n_-fluorophore2, where n=2-7 and fluorophores formed an interacting pair of energy donor and acceptor engaged in Förster resonance energy transfer 19. As an example of such design, the structure of FAM-**FFFFFF**TCTAAATCACTATGGTCGC**FFFFFF**-Dabcyl carrying six **F**-base pairs included in the stabilizing “stem” is shown in Table 2 (compound **9)**. Two related synthons **VIIIb** and** VIIIc** (Table 1) 20, 21 were obtained by using identical synthetic steps and were used for the synthesis of specially designed probes for ^19^F NMR spectroscopy (Table 2, compound **10a** and** 10b**) with 2-(hydroxymethyl)-2-[3,5-bis(trifluoromethyl)benzamido]ethyl- or 3,5-bis(trifluorometyl)benzyl-groups linked to the 5'-phosphate.

**Scheme 6:**
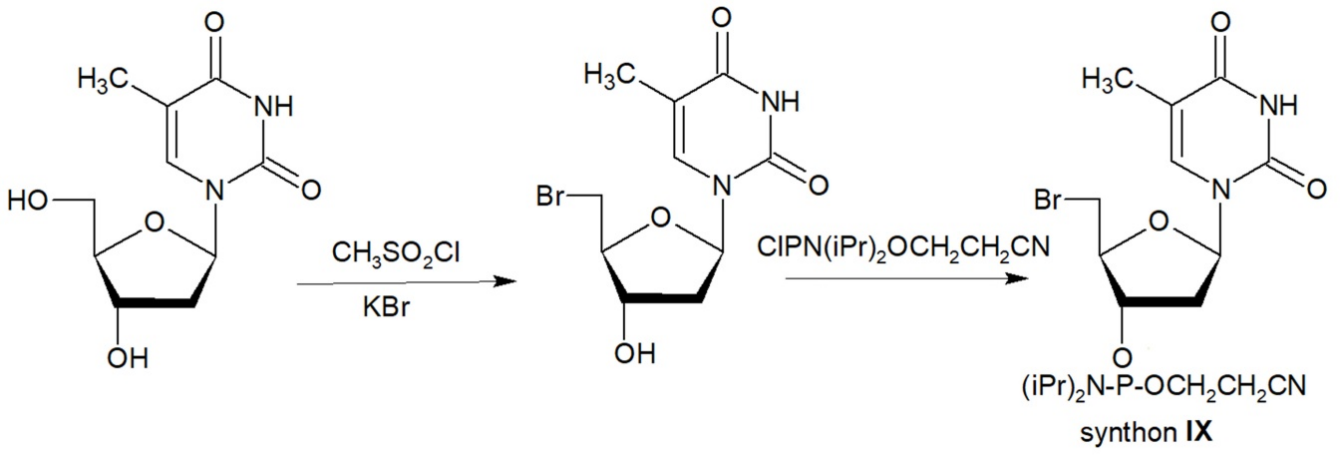
Synthon **IX** was synthesized by thymidine bromination in two steps followed by 3'-O-phosphitylation.

The second approach previously used for the synthesis of MF-ONs included the introduction of monomeric units that allowed incorporating reactive groups into the oligonucleotide chain for subsequent conjugation with fluorinated compounds. 5'-Bromo-5'-deoxythymidine-3'-phosphoramidite (**IX**) and commercially available 5'-carboxy-modifier C10 (**X**, Table 1) were introduced as reactants at the final step of solid phase oligonucleotide synthesis. The synthon **XI** 5-[3-aminoprop-1-ynyl]-2'-deoxyuridine-5'-O-triphosphate was used for incorporating modified nucleotides into ON by using primer extension method while synthon **IX** allowed click-chemistry modifications (see Scheme 8). The synthesis of synthons **IX** (Table 1) was accomplished by following the **Scheme 6** 18**.**

**Scheme 7:**
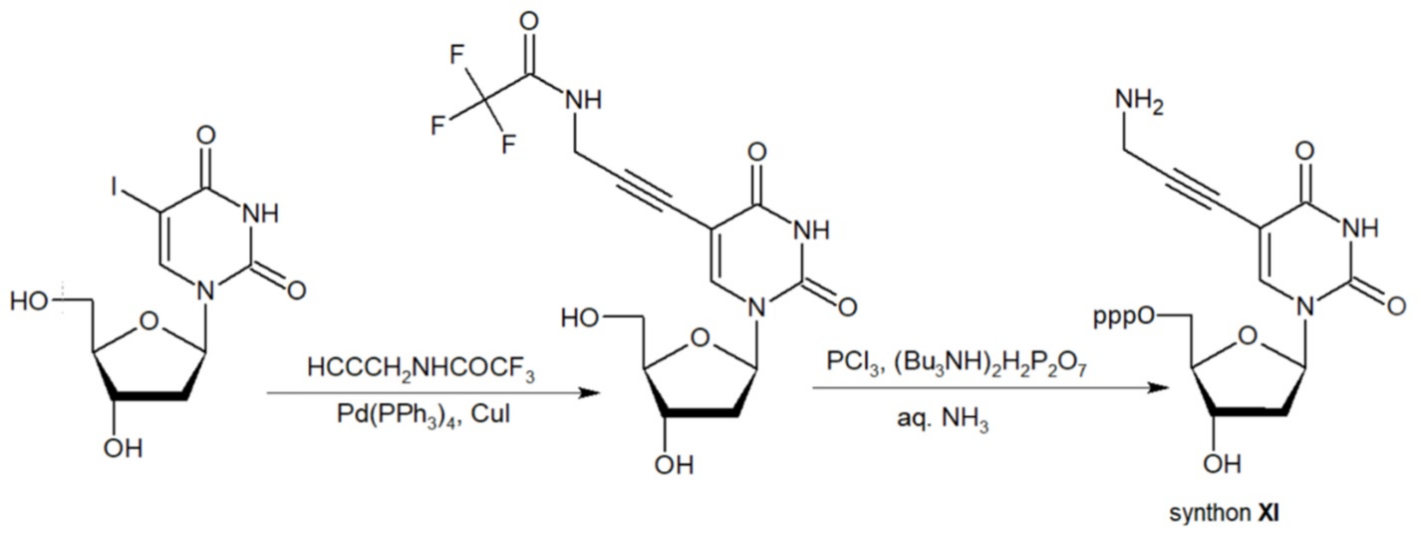
The synthesis of synthon **XI** starting from palladium-catalyzed coupling of 5-iodo-2'-deoxyuridine with an alkyne.

Synthon **XI** (Table 1) allowed introducing primary amino groups 5-(3-aminoprop-1-ynyl)-2'-deoxyuridine units into oligonucleotide chain and its synthesis was accomplished 23 by following the **Scheme 7**.

### MF-ON synthesis using post-synthetic modifications

**Scheme 8:**
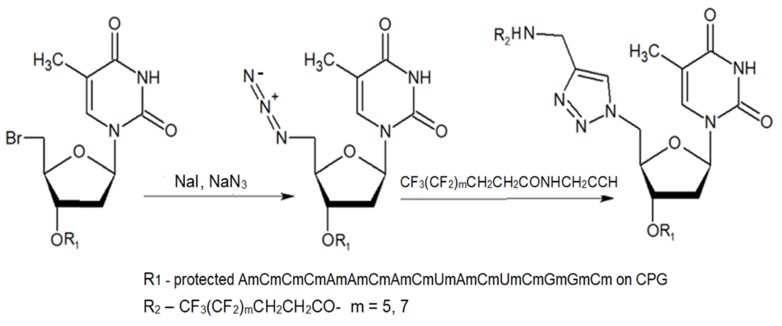
The synthesis of MF-ON** 11a,b** accomplished via a 1,3-dipolar cycloaddition involving an azido ON intermediate and propargylated fluorocarbon chains.

ON and their analogs were conjugated with appropriate fluorine-containing compounds by using standard approaches such as: 1) click chemistry, 2) activation in the presence of HBTU/HOBt, HCTU, 3) using activated esters such as N-hydroxysuccinimides, or 4) by haloacetyl reaction with thiol. The conjugation was achieved either immediately after synthesis on the column, or after purification. The synthesis of MF-ON **11a** and **11b** (Table 2) was achieved by using a phosphoramidite synthon **IX,** which was coupled to a 17-mer oligonucleotide using a solid support synthesis via a 3'- to 5'- elongation approach 22. The target compounds were synthesized according to the **Scheme 8**.

**Scheme 9:**
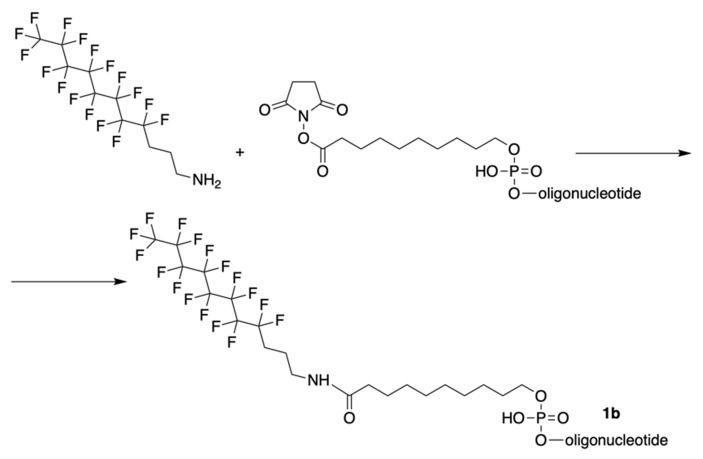
The synthesis of oligonucleotides with 5'-modified group as in MF-ODN **1b** from properly protected ONs and linking to CPG by conjugation with 3-(perfluorooctyl)propylamine via activated carboxy groups.

The synthesis of MF-ONs via linking 9-[3-(perfluorooctyl)propylcarbamoyl]nonyl (FCN) residues was achieved 10 immediately after the synthesis of oligonucleotide with commercially available 5'-carboxy-modifier C10 (**X**, Table 1), which was introduced at the last step according to the **Scheme 9**.

As a consequence, four different modified 21-mer ONs with FCN groups at 5'-end were obtained (e.g. **1b** , see Table 2).

**Scheme 10:**
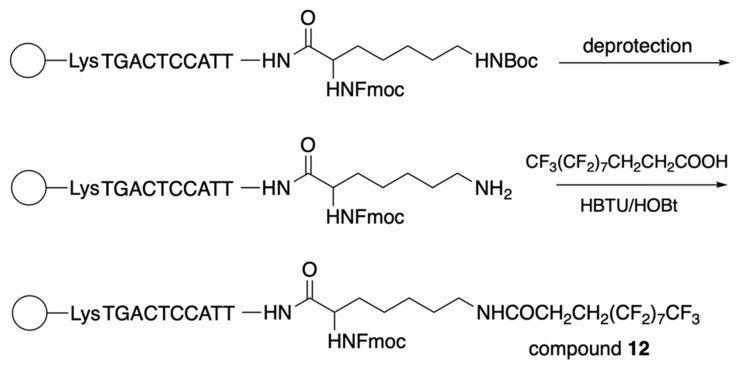
The final steps of compound **12** synthesis: deprotection of the ω-amino group and coupling of the resultant compound with 2H,2H,3H,3H-perfluoroundecanoic acid using HBTU/HOBt as a coupling reagent under microwave irradiation.

The synthesis of 3-(perfluorooctyl)propanoyl chain-conjugated peptide nucleic acids (PNA) 24 (compound **12**, Table 2) was performed as follows: after the addition of the last PNA monomer, N^α^(Fmoc)-N^ε^(*t*Boc)-Lysine was coupled to the PNA at the N-terminus and further by following the **Scheme 10**.

**Scheme 11:**
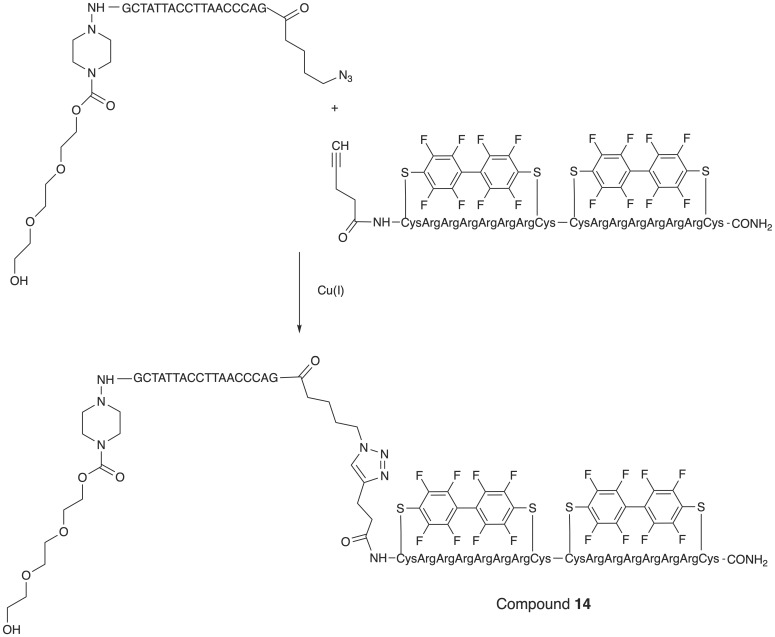
The last step in the synthesis of compound **14:** copper-catalyzed cycloaddition of specially modified PMO *GCTATTACCTTAACCCAG* and bicyclic peptides.

Fluoroalkyl phthalocyanine-oligonucleotide bioconjugates (as example, compound **13**, Table 2) were obtained by using solid-phase synthesis by combining the carboxy-derived perfluorophthalocyanine with 5'-aminoalkyloligonucleotide-bound resin by a coupling reagent HCTU: O-(1*H*-6-chlorobenzotriazole-1-yl)-1,1,3,3-tetramethyluronium hexafluorophosphate 25. Synthesis of a conjugate of phosphorodiamidate morpholino oligonucleotides (PMO) with perfluoro bicyclic cell-penetrating peptide (compound **14**, Table 2) was accomplished 26 according to the **Scheme 11**.

**Scheme 12:**
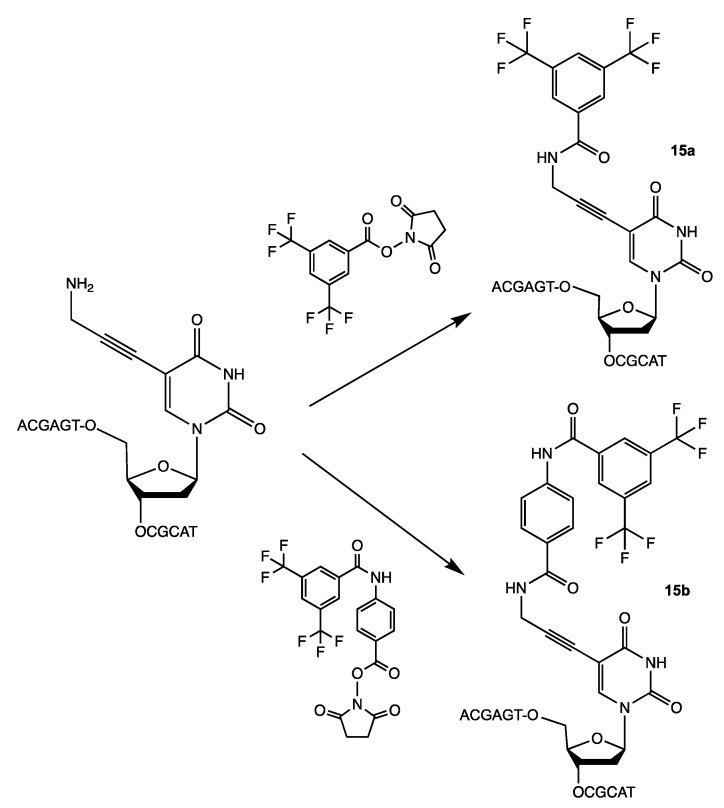
Final step in the synthesis of compounds **15a** and **15b**.

N-hydroxysuccinimides of 3,5-bis(trifluoromethyl) benzoates were used for the synthesis of oligonucleotides with ^19^F-labeled nucleobase 27. Purified oligonucleotides with introduced 5-[3-aminoprop-1-ynyl]-2'-deoxyuridine unit were coupled with N-hydroxysuccinimide ester of 3,5-bis(trifluoromethyl)benzoic acid or 4-[3,5-bis(trifluoromethyl)benzamido]benzoic acid to yield compounds **15a** and **15b** (Table 2), **Scheme 12**.

**Scheme 13:**
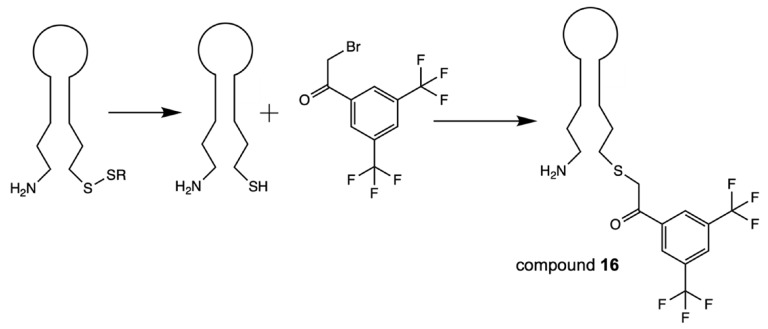
Synthesis of compound **16** via the reduction of corresponding disulfide followed by haloacetylation.

Recently, the conjugation of 3,5-bis(trifluoromethyl)phenyl group that results in the “magnetically active” oligonucleotide beacon was performed by linking 2-bromo-1-[3,5-di(trifluoromethyl)phenyl] ethan-1-one to hairpin oligonucleotide through the haloacetylation of the corresponding thiol 28 (**Scheme 13**), compound **16** (Table 2).

## Properties of oligonucleotides modified with MF groups

In general, because of strong fluorous effect the properties of ON modified with MF groups/ modifiers are expected to differ from those of ONs bearing hydrophobic groups (e.g. acylated with fatty acids or derivatives of cholesterol) 22. Those properties stem from the combination of the fluorous effect, i.e. the interaction between individual fluorine atoms that may result in supramolecular structures, and a combination of hydrophobic/lipophobic effects of MF groups/modifiers that may lead to an exclusion of those groups from water milieu. The likely outcome of this interaction is transient adsorption of supramolecular structures on hydrophobic surfaces which in similarity with various surfaces modified with hydrophobic residues 30 may result in transient interactions of hydrophobic domains in the membranes with the hydrophobic bases of ONs.

**Figure 1:**
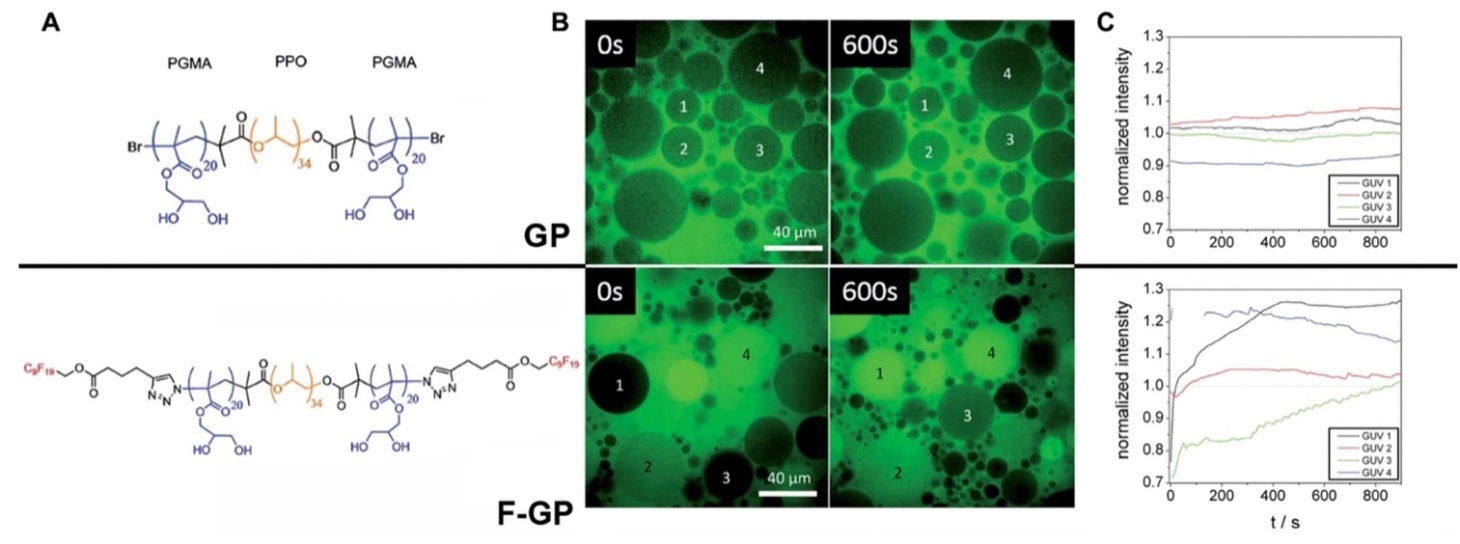
Permeation into the membranes due to “fluorous effect”. **A**- structures of GP and F-GP copolymers containing hydrophobic (yellow), hydrophilic (blue) and fluorophilic (red) building blocks. **B** - confocal laser scanning microscopy images of solutions containing water-soluble AlexaFluor 488 dye and 1) POPC giant unilamellar vesicles (GUVs) + GP (8 mM), upper row or POPC GUVs + F-GP (7.1 mM), lower row at 0 and 600 s after the injection of the GUVs in the solution of the dye and copolymer; **C**- fluorescence intensities measured within selected POPC GUVs after the addition to AlexaFluor 488 dye solution containing (**A**) no additive, (**B**) GP (8 mM) and (**C**) F-GP (7.1 mM) normalized by the average fluorescence intensity of the full frame image. The numbering of the GUVs corresponds to the numbering in panel **B**. Adapted with permission from 33, copyright 2014, The Royal Society of Chemistry.

Furthermore, the lipophobic nature of MF modifiers may prevent the incorporation of MF compounds into the inner lipid core of membrane bilayers potentially creating defects in membranes that may promote MF-ON transport via model membranes and plasma membranes of cells. Consistent with such hypothesis is the observation of the self-assembly of MF-ON at the cellular exterior with the formation of resultant nanosized aggregates. Other non-ON amphiphilic fluorinated compounds were also found to form micelles that interacted with the surface of membranes 31. It was hypothesized that such nanosized aggregates would be taken up by cells via energy-dependent endocytosis pathway 6. It is also possible that poor miscibility between fluoroalkyl and phospholipids and resultant MF-ON exclusion from lipid membranes leads to improved cytotoxicity profiles 32. The results of studies that used polymers containing perfluoroalkyl or semifluoroalkyl groups are useful in explaining how MF tails help ONs to traverse lipid membrane barrier 33, 34 (Fig. 1A). The authors established that MF chains can function as anchors inducing phase separation in the membranes thereby causing the formation of defects that allow penetration of low molecular weight water-soluble molecules and simultaneously promote translocation of larger polymers through the membranes 34 (Figure 1B, C). Therefore, it is possible that unusual properties of MF-ON that drive aggregation and dis-aggregation of MF-ON assemblies are responsible for remarkable ON-membrane traversing process. These and other studies reporting the use of fluorous effect suggest that instead of direct incorporation of fluorine atoms into the structure of ON or other molecules the fluorination of carrier molecules can assist in more efficient delivery of non-modified cargo molecules into the cells. Over the last five years several groups successfully used fluorination for *in vitro* and *in vivo* delivery of native (i.e. non-modified) DNA expression vectors 35-37, as well as peptides and proteins 32, 38, 39 by using low-molecular weight or high-molecular weight branched positively charged polymeric carriers (reviewed in 40). The delivery of hydrophobic low molecular weight compounds *in vitro* using theranostic multifluorinated nanogel carriers has also been recently reported 41. It is highly likely that in the case of MF-ON the efficacy of multi-fluorinated carriers could be further improved 10. The accumulated evidence regarding the fluorous effect on ON behavior the use of MF-ON probes and MF-ON based materials for probing interactions with various biological targets are summarized below.

### The influence of MF on stability of ON duplexes

The ability of MF-ON to form duplexes due to Watson-Crick base pairing can be potentially strengthened or, conversely, substantially weakened by covalent modification of individual oligonucleotides forming a duplex. The MF moieties are no exception and the formation of duplexes was studied in several reports that focused on MF-ON. For example, an introduction of a single terminal MF group has only a very slight influence on thermal stability (1-1.5 degree) of the corresponding duplexes 10, 16. An overall effect resulting in a decrease of MF-ON duplex melting temperature (T_m_) with MF moiety in the 5-position of cytosine has been reported 18.

GCAATCC*GGTAGCGTTAGG-CCATC, where C* is 5-[CF_3_(CF_2_)_n_CH_2_CH_2_]-C, n=1,5,7.

The measured T_m_ values showed a decrease as the length of the MF (from 2 to 8) increased, suggesting that MF groups could cause interference with base pairing. Nonetheless, the T_m_ values of duplexes were all in the range of 53-58°C, indicating that stable double helix structures were formed for all double-stranded DNAs containing MF groups.

**Figure 2:**
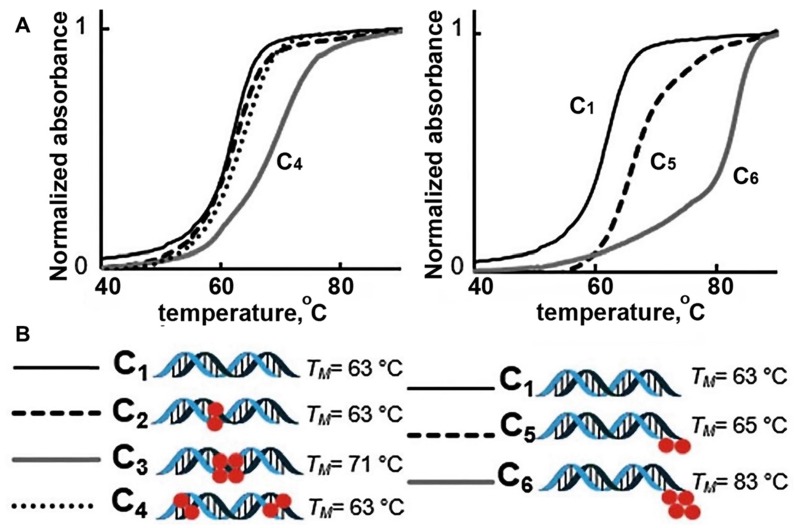
Representative melting temperature curves of MF-containing DNA 19-mer duplexes. Red spheres represent the MF- modification. Left: Internal modifications. Right: External modifications. Adapted with permission from 16, copyright 2016 The Royal Society of Chemistry.

The introduction of multiple MF groups into the composition of a 19-bp ON was shown to change T_m_ of resultant duplexes significantly, e.g. T_m_ of duplexes C3 and C6 16 (Figure 2). In these duplexes postulated interaction of single MF groups on complementary ONs (marked by red on Figure 2) did not show any substantial change of T_m_ values suggesting that the stability of duplexes C2, C4 was similar to the duplex without MF groups, i.e. C1. However, T_m_ value determined in the case of duplex C3 and C6 were much higher than Tm of C1 suggesting very strong interactions and duplex stabilization. The possible explanation is that the observed Tm increase was a result of the proximity of two MF groups on each of ON (MF-ON **6e** and **6h** in Table 2), which results in strong multiple fluorine-fluorine interactions. Thus, when two adjacent MF units were incorporated into the interior portion of each ON strand using synthon **V** (Table 1), a dramatic T_m_ increase of about 8°C followed. Importantly, if these two modifications within each strand are not adjacent (C4), the T_m_ increase was much less pronounced. The authors proposed that MF chains thus likely merge into a “fluorous” environment, avoiding unfavorable interactions with water and leading to significant stabilization of duplexes. If MF groups were positioned at the termini of the ONs forming the duplex, a single modification on each ON strand (C5) increased T_m_ by 2°C while an increase of 20 °C was observed in the case of two modifications on each strand (C6). Such dramatic increase of T_m_ can only be explained by a formation of higher order structures from individual duplexes. i.e. it is possible that different duplex dimers (C5-C5 and C6-C6) could be formed after MF modification. There is a potential formation of “MF clips” (indicated as **•**) that in theory participate in joining individual duplexes differently, as shown below:

5' TTTTTCAGTTGACCATATA-MF-3'•3'-MF-ATATACCAGTTGACTTTTT 5'

3' AAAAAGTCAACTGGTATAT-MF-5'•5'-MF-TATATGGTCAACTGAAAAA 3'

5'TTTTTCAGTTGACCATATA-MF-MF-3'•5'MF-MF-TATATGGTCAACTGAAAAA 3'

3'AAAAAGTCAACTGGTATAT-MF-MF-5'•3'MF-MF-ATATACCAGTTGACTTTTT 5'

C6**•**C6 MF-ON dimer may potentially exhibit properties of a pseudo continuous 38-mer. By using available on-line resource it is easy to estimate that T_m_ of a non-modified 38-mer ON with the same sequence as two C6 will be higher than T_m_ of 19-mer ON duplex by approximately 20 degrees 42. Thus, it appears that fluorous effect due to multiple MF moieties introduced by using synthon **V** or a branched synthon **IV** (Table 1) could provide DNA duplex stability increase across the range of temperatures, and might be useful for assembly of various supramolecular structures from individual duplexes.

### MF-ON as imaging and spectroscopic probes

**Figure 3:**
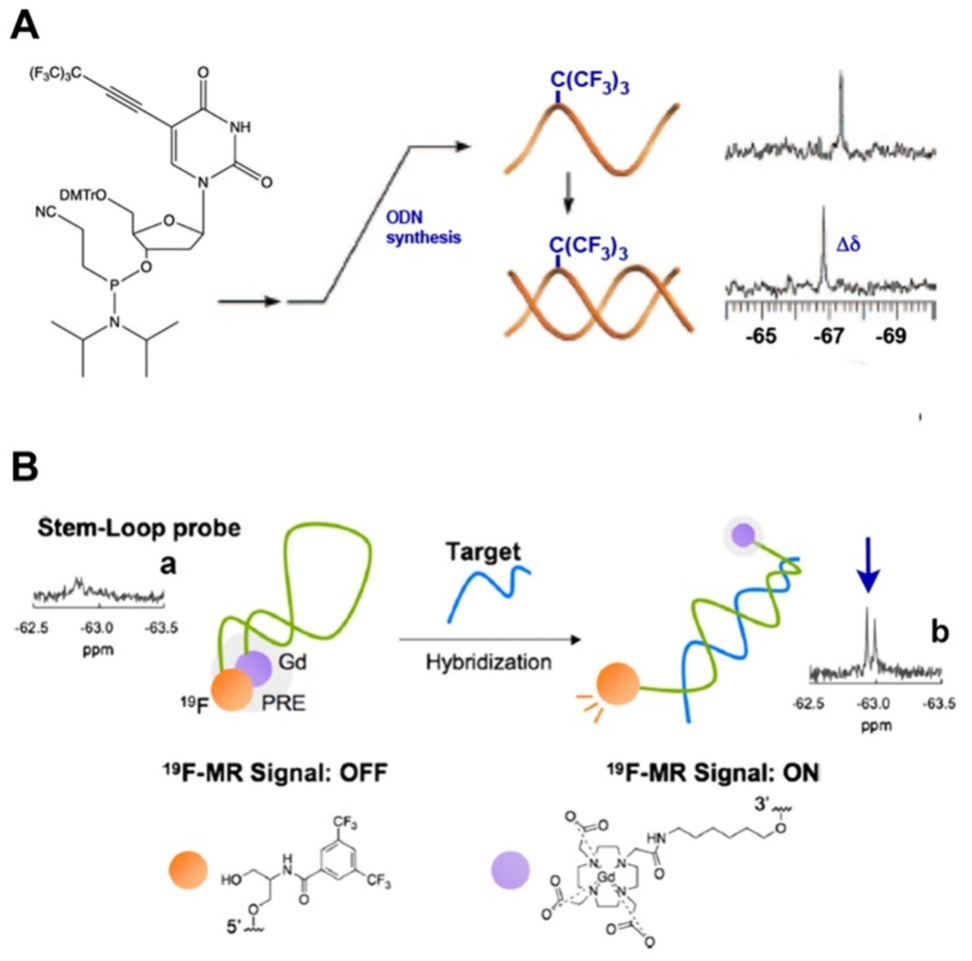
** A** - a nucleoside carrying a tert-butyl MF group that was incorporated in ODN. Transition from single strand to duplex was monitored by ^19^F NMR spectroscopy at micromolar concentrations of oligomers that showed a chemical shift change (Δδ), demonstrating the sensitivity as an NMR reporter nucleoside, adapted with permission from 17, copyright 2008, American Chemical Society; **B**- a schematic drawing of the mechanism of target nucleic acid detection using stem-loop typed oligonucleotide probe with incorporated MF group and Gd-DOTA at the opposite termini of the probe. ^19^F NMR spectra and signal intensity of the probe in the presence of various oligonucleotides. (a) Probe alone; (b) + Kras Mut; the arrow points to ^19^F NMR signals of the bis(trifluoromethyl)benzene moiety. Adapted with permission from 20, copyright 2019 Elsevier B.V.

It is commonly assumed that both NMR spectroscopy and MRI could take advantage a negligible ^19^F background signal due to the fact that soluble molecules containing fluorine atoms in living organisms are present at very low concentrations and that the great majority of those are technogenic. Moreover, the wide chemical shift range of various ^19^F nuclei is also advantageous in that it enables the design of various sensors and facilitates imaging of several molecular species at once 43. The incorporation of single fluorines in nucleic acids has been used for decades for the purpose of structural studies involving nucleic acids interactions cognate biological targets such as complementary ON and DNA-binding proteins 43-45. In the case of MF-ON probes the presence of multiple fluorines improves sensitivity and provides unique spectroscopic signatures. For example, using the nucleoside synthon VIII (Тable 1) as a precursor of an NMR reporter nucleoside 17 MF-ONs containing perfluorinated *tert*-butyl group (nine magnetically equivalent fluorine atoms total per molecule) were synthesized and used for spectroscopic monitoring of a duplex formation to investigate the transition from single strand to duplex achieving micromolar sensitivity to ONs. The incorporation of multiple magnetically equivalent fluorine atoms into the ON's composition resulted in a chemical shift of -67.36 ppm (relative to CCl_3_F) in a single-strand ON, and with the addition of the complementary strand a new peak at -66.91 ppm was observed, which signified the formation of the ON duplex 17 (Figure 3A). A sensor DNA molecule with stem-loop configuration that was tuned to interaction with a complementary ON by ^19^F MR signal was proposed in 20. The probe was based on the paramagnetic relaxation enhancement (PRE) effect due to interaction with Gd-DOTA chelate linked to the 3'-terminus of the ON probe. The resultant molecular beacon carrying bis(trifluoromethyl)benzene moiety on the 5'-end was able to report on a formation of a duplex with target nucleic acid as a result of ^19^F MR signal “turn-on” with sequence specificity as demonstrated in the case of synthetic *Kras* ON sequence carrying mutations 20 (Figure 3B). A similar design of “responsive” ^19^F hairpin-like probe containing modifications at both of the termini of the hairpin has been recently reported in which the fluorinated group served as a reporter 28. The magnetically equivalent fluorine atoms (a result of a modification of the ON's terminal thiol with a MF residue) served as reporters similar to the design of the beacon initially suggested in 17. When this hairpin was not bound to the complementary target the authors observed a broadened ^19^F NMR peak (turned “off”) due to PRE effect of the nitroxide radical. The ^19^F NMR peak of the beacon was “recovered” in the event of beacon ON sequence hybridization to a complementary ON due to physical separation from the nitroxyl radical 28. Therefore, in addition to widely-used fluorescent labeled ON beacons PRE-effect based probes may be potentially used for detecting complementary ON _structures_ as well as sequence mismatches. If the delivery of excitation light becomes a problem because of the turbidity of the medium then MR spectroscopy and imaging become attractive alternative to fluorescent imaging.

### The ability of MF-ON to form nanoparticles

**Figure 4:**
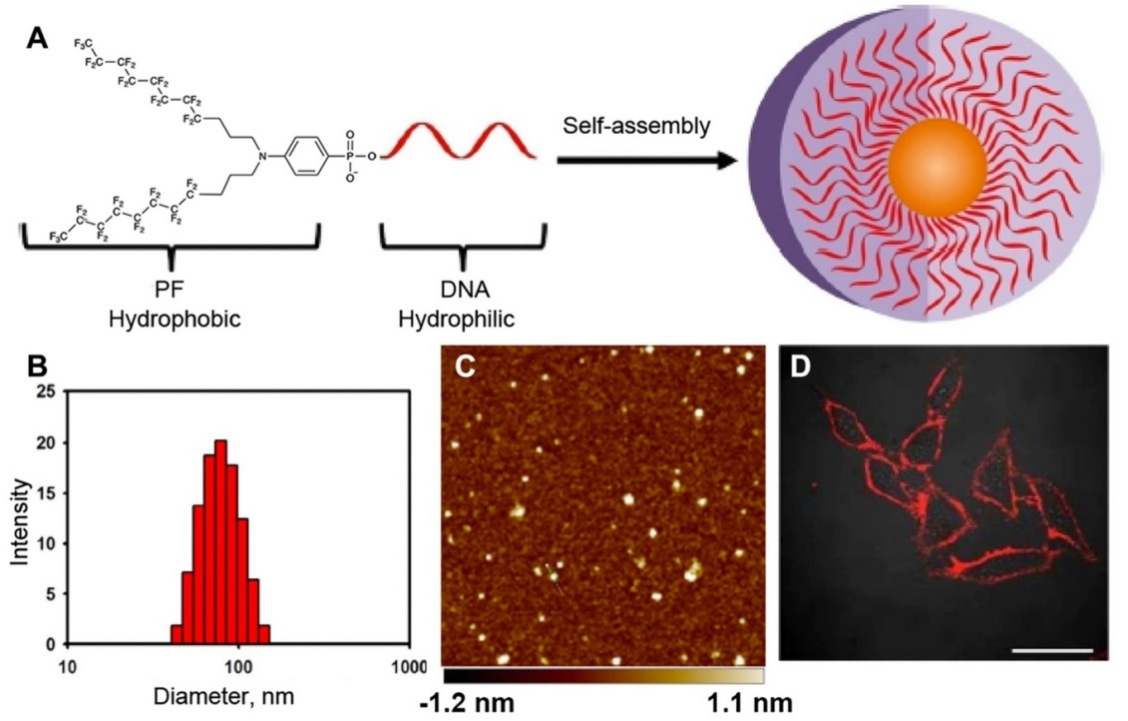
** A** - Bis(perfluorooctylpropyl)-DNA Micelle (PFDM) containing a hydrophobic perfluorocarbon core and a hydrophilic oligonucleotide corona. **B**- dynamic light scattering (DLS) of the self-assembled PF-T_15_ micelles; **C** - atomic force microscopy of self-assembled PF-T_15_ micelles on a mica surface; **D** - HeLa cells treated with TAMRA-labeled PFDM (200 nM) and subsequently examined by confocal microscopy. Adapted with permission from 15, copyright 2018, American Chemical Society.

The behavior of MF-ON in water solutions depends on the following factors: 1) the structure of MF component, 2) the chemical nature of ON segment (i.e. whether ON is comprised of DNA, RNA, ON analogs like PNA, PMO or their combinations), 3) the length of ON, 4) the net charge, 5) media components (i.e. ionic strength, temperature, the presence of cations of bivalent metals). The assembly properties of MF-ON with particular single or multiple MF groups was previously reported in some detail in 16. In that study, MF groups were introduced into oligonucleotide chains or their termini by using synthon **V** (Table 1). The examples of the structure of several ONs and their duplexes are shown in Table 2 (MF-ON** 6a-6h**). MF-ON **6a** with just one single terminal perfluorooctyl MF group does not self-associate and form micelles in water even after the addition of magnesium cations (Mg^2+^) while MF-ON **6b** does not aggregate in pure water but the addition of Mg^2+^ triggers self-assembly into nanoparticles. Upon the addition of Mg^2+^, MF-ON **6c** forms micelles with 4 or 5 MF-ON monomeric units, which undergo a significant radius decrease within one minute after adding (from approximately 8 nm to 6 nm). Mg^2+^ likely cause neutralization while assisting in formation of salt bridges between the negatively charged ON phosphates. The resultant decrease of the repulsive electronegative interactions between the ON DNA strands and MF components promotes the self-assembly into nanoparticles. The analysis of such micelles by using denaturing polyacrylamide gel electrophoresis in 7 M urea showed that in ON with four and five MF moieties very strong self-assembly interactions occur and the resultant material is not able to penetrate into the gel matrix. In a similar study it has been demonstrated that an ON with terminal 4-(bis[3-(perfluorooctyl)propyl]amino)phenyl groups (MF-ON** 5a**, Table 2) promote a spontaneous self-assembly into the micelle-like structures 15 (Figure 4). The addition of acetone (v/v = 1:1) resulted in the dissociation of those micelle-like structures. The elongation of oligothymidine chain from n=15 to n=45 diminished the stability of micelles. The hydrodynamic diameter of micelles in PBS was 57.8 nm with a polydispersity index of 0.382 as measured by DLS. However, the visualization of morphology by using atomic force microscopy showed a layer of uniform spherical particles with smaller diameters of 22.9 ± 3.4 nm. The linking of MF groups to PNAs leads to the formation of smaller size nanoparticles (100-250 nm) from control non-fluorinated (C11-hydrocarbonundecanoyl)-PNAs compared to the larger size nanoparticles (500 nm), which according to the authors may improve the efficiency of PNA penetration into the cells 24.

Therefore, as follows from several reports published so far the MF moiety does impart a tendency to promote self-assembly of MF-ON into micelle-like structures. If the goal of fluorination is in improving delivery into the cells it appears important to identify each particular oligonucleotide with the corresponding MF modification that facilitates the crossing of the membrane barrier while avoiding the formation of very stable self-associated micelles that would not permeate into or dissociate within the cells.

### The stability of MF-ON against nuclease-mediated hydrolysis

Multifluorinated groups can potentially provide additional benefit of increasing stability to DNA hydrolysis by nucleases due to the protein-repelling properties of MF group that are able to protect short non-modified (native) siRNA-based duplexes from degradation by linking those MF groups to carrier molecules 46. However, for increasing stability MF- modifications may be introduced into the ONs directly thereby eliminating the need in carrier molecules. To compare the stability of MF-containing and control duplexes, a non-modified 19-mer duplex and MF-ON conjugates C1-6 (Figure 2) were exposed to fetal bovine serum (FBS) at 37°C 16. As a result, C2 duplex with a single MF group on each ON showed better nuclease resistance than duplex C1. Interestingly, two adjacent modifications enhanced the half-life of the duplex in FBS by a factor of 3. With two modifications at the end of each DNA strand, C6 showed impressive nuclease resistance properties since approximately 50% of the initially detected ON were still present after 24 h incubation in contrast to only 3% in the case of C1. Thus, perfluorocarbon modifications could increase DNA stability very significantly and simultaneously improve the resistance to nucleases.

**Figure 5:**
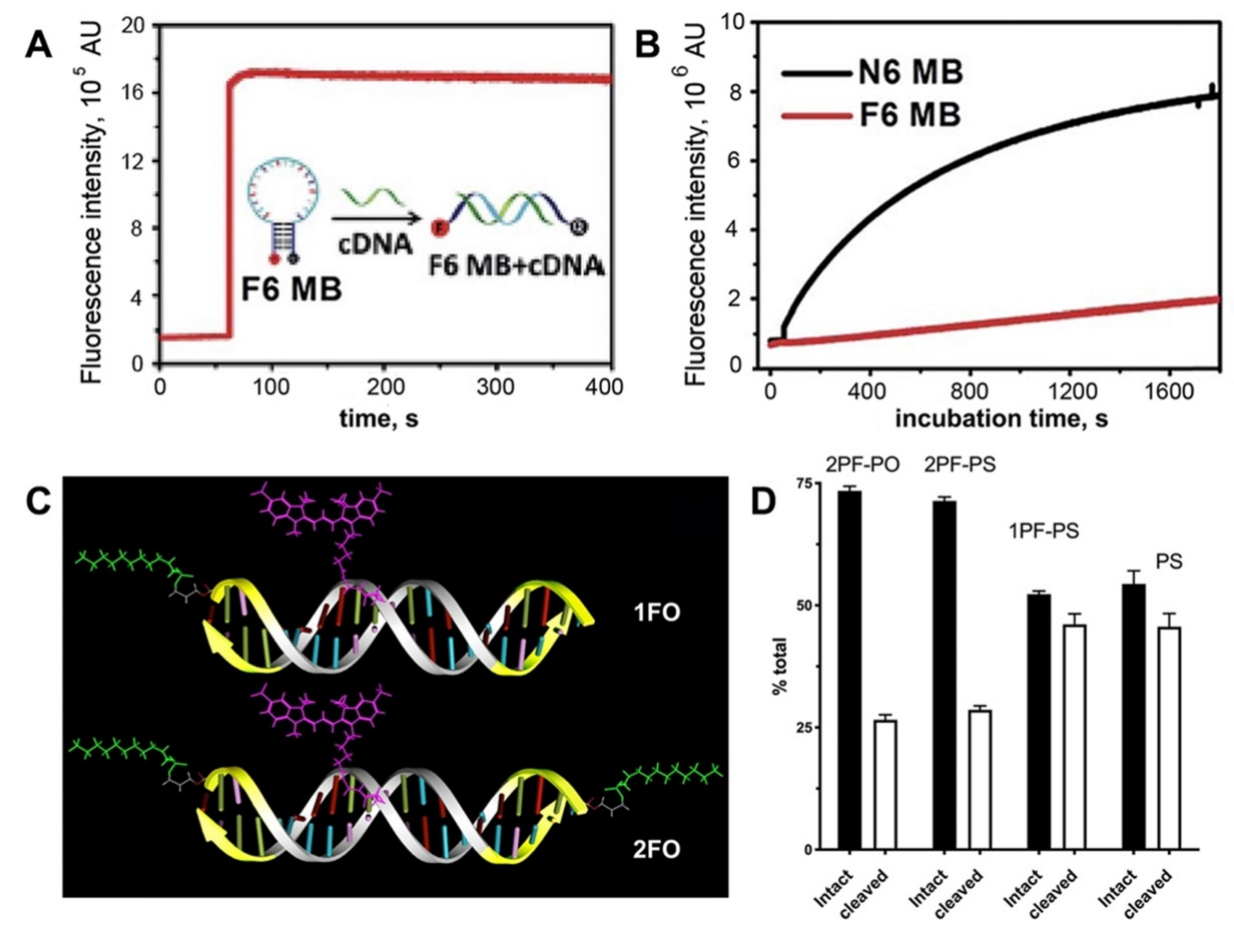
Stability of MF-ODN. **A**- fluorescence intensity kinetics of F6 MB (a molecular beacon carrying six F-base pairs) treated with complementary DNA **B**- fluorescence intensity change over time in the solutions of F6 MB (red trace) and N6 MB (black trace) after treating with 0.25 U/mL DNase I, adapted with permission 19 copyright 2017, The Royal Society of Chemistry; **C**- a scheme showing major structural elements of fluorescent, Cy3-labeled oligonucleotide duplexes (ODND), containing either one 5'-linked perfluorooctylpropyl group (1FO-ODND-Cy3) or two FO groups (2FO-ODND-Cy3). Fluorinated residues are shown in bright green, Cy3 fluorophore is shown in magenta. Yellow - phosphorothioates in hybrid (PS) ODNs; **D**- quantification of intact and degraded fractions of ODND as determined by using ROI measurements of integrated band intensity after gel electrophoresis on 15% polyacrylamide gel stained with SybrGold, adapted with permission from 10, copyright 2017, Ivyspring International Publisher.

A different design containing ON duplex elements, i.e. molecular beacons with multiple terminally linked **F-**bases were also tested in nuclease stabilization assays 19. It was shown that these beacons have increased stability due to the fact that fluorinated base pairs cannot be recognized by endonucleases (Figure 5A,B). Consequently, the replacement of natural base pairs by **F**-bases pairs makes modified ON more resistant to degradation. Therefore, it is potentially possible that the formation of the micelle-like structure could improve the stability of DNA molecules 15.

Previously reported near-infrared fluorescent ON duplexes were modified by one or two MF groups at 5'-ends by using 3-(perfluorooctyl)propyl residues 10. These duplexes were designed as imaging probes for STAT3 binding for deep tissue imaging. The modification was achieved by using the synthon **Ia** (Table 1) 10. The stability of four obtained ON duplexes:

** Cy3**

5'** -AGCC**|ATTTCCCGTAAAT**CTCT**-3'

3'- **TCGG**TAAAGGG|CATTTA**GAGA**-5'

** Cy3**

5'-CF_3_(CF_2_)_7_(CH_2_)_3_pAGCC|ATTTCCCGTAAATCTCT-3'

3'-TCGGTAAAGGG|CATTTAGAGAp(CH_2_)_3_(CF_2_)_7_CF_3_-5'

5'-CF_3_(CF_2_)_7_(CH_2_)_3_p**AGCC**AT|TTCCCGTAAAT**CTCT**-3'

3'-**TCGG**TAAAGGG|CATTTA**GAGA**p(CH_2_)_3_(CF_2_)_7_CF_3_-5'

**Cy3**

5'-**AGCC**|ATTTCCCGTAAAT**CTCT**-3'

3'-**TCGG**TAAAGGG|CATTTA**GAGA**p(CH_2_)_3_(CF_2_)_7_CF_3_-5'

** Cy3**

in the presence cell culture-conditioned medium was analyzed by using agarose electrophoresis (see examples in Figure 5C). All four duplexes were binding to medium components regardless of the presence of MF groups. After incubating samples at 37^o^C it has been determined that all MF-ON and control ON duplexes showed at least some degree of degradation and association with the cell culture media proteins (between 7.5% and 12% of total ON). After incubation the degradation was at the level of 33-35% for MF-containing ON with three phosphorothioate bonds (Figure 5 C, D) at each terminus. In the absence of MF groups the degradation of ON exceeded 50%, which indicated at least some protective effect of MF groups in the case of a mix of nucleases in the cell culture medium.

### Permeation of MF-ON and their analogs into eukaryotic cells and lysosomal escape

It has been reported that ON and their synthetic analogs such as locked nucleic acids modified with phosphorothioates (i.e. gap-mers) could be delivered into the eukaryotic cells in the absence of any transfection reagents via mammalian ortholog of SID-1 proteins (SIDT-2) due to the process called gymnosis 47, 48. At present it is unclear whether gymnosis is involved in transmembrane transport of MF-ON bearing bulky fluorinated groups. However, the consequences of fluorine-fluorine interactions may include the formation of supramolecular structures and particle compaction which may facilitate cellular internalization of these fluorine-containing particles 22, 24. The efficient uptake in the absence of transfection-promoting agents usually requires either lipid or fluorinated chain conjugation to the ONs. Initial experiments with MF-ON have shown that similarly to hydrocarbon-conjugated ON (L-ON) the linking of MF groups improves intracellular uptake of nucleic acids. The observed toxicity to cells of MF-ON was negligible and cellular internalization of MF-ON without any transfecting reagents was efficient and was not affected by the presence of serum 22.

Unlike classic ONs fluorescent-labeled PNAs modified with a single perfluoroundecanoyl chain showed 2.5 -3 times higher uptake efficiency in NIH 3T3 and HeLa cells compared to their hydrocarbon-modified undecanoyl PNA counterparts 24, 49. Strong 10-fold increase of mean fluorescence intensity was noted and 2- times (HeLa) and 3-times (3T3 cells) higher number of fluorescent cells were detected in the case of MF-PNA compared to hydrocarbon-modified PNA. Similarly to the results reported in 16, the linking of perfluorinated moieties to PNAs resulted in the formation of smaller size nanoparticles (2-times, mean diameter- 250 nm) compared to larger size nanoparticles formed by non-fluorinated PNAs. This may explain better efficiency of cell uptake and/or trans-membrane transport and these results may provide a rationale for engineering PNAs with improved cell delivery properties.

**Figure 6:**
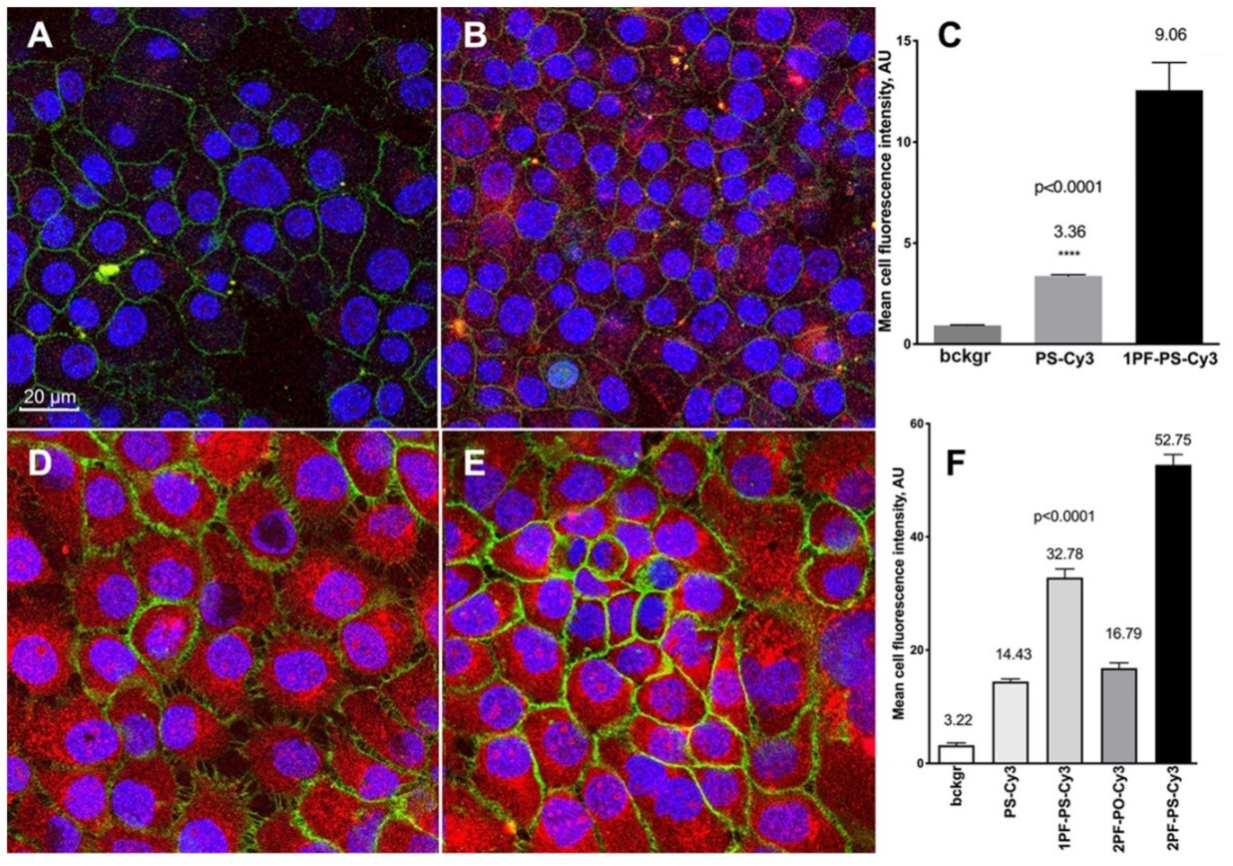
Confocal microscopy and quantification of cellular uptake of the MF-modified ON duplexes. Cy3-labeled ON duplexes (0.5 µM) modified with 5'-linked MF groups were incubated for 18 with the cells. **A**, **B** - confocal multichannel fluorescence microscopy of insulinoma INS-1 cells after an overnight incubation with PS-Cy3 (**A**) or 1PF-PS-Cy3 (**B**) ON duplexes. **D**,**E**- confocal microscopy of human squamous cell carcinoma A431 cells after incubating with 1PF-PS-Cy3 (**D**) or 2PF-PS-Cy3 (**E**). Blue channel: DAPI. Green channel: anti-EGFR-AF488 (A431 cells) or AF488-NHS (INS-1). Red - Cy3 (ON). **C**, **F** - quantification of the uptake using mean fluorescence intensity of Cy3 in individual cells (**C**- INS-1; **F**- A431). Column statistics and P values were determined by using non-parametric Mann-Whitney test, n=30-60 cells. Adapted with permission from 10, copyright 2017, Ivyspring International Publisher.

The incorporation of MF-ON micelle components into cell membranes was investigated in 15. The components of those micelles included MF-ONs synthesized by using a branched MF -synthon IV (Table 1) while the opposite terminus of ON carried a pH-insensitive rhodamine dye (TAMRA) to avoid the influence of cellular compartments with altered pH environments. T lymphoblast-like cells (CCRF line) were subjected to a short-term incubation in the presence of TAMRA-labeled MF-ON or TAMRA-labeled control ON. Flow cytometry of cells showed large TAMRA fluorescence intensity shift (137 times) of the cells treated with MF-ON while only minimal fluorescence signal was detected in the cells treated with MF- free ON probes. This result was explained by the effect of introducing a MF group that greatly facilitated the interaction of DNA macromolecules with live cells. The intracellular location of MF-ON was determined by using confocal microscopy which showed that MF-ON treatment resulted in a bright TAMRA signal localized on the surface of the cells. TAMRA-labeled MF-ON was also anchoring at the membrane of adherent HeLa cells. The authors speculate that ON micellar structures tend to dissociate and the monomeric MF-modified units undergo a spontaneous insertion into the plasma membrane due to local alterations of hydrophobic interactions within the membrane. Сysteine arylation chemistry was used to introduce multiple fluorinated groups into arginine-rich peptide conjugated to PMO to modulate their ability to induce exon skipping in reporter HeLa cell line where ON analogs corrected EGFP splicing and restored fluorescence of the reporter gene 26. These bicyclic peptides exhibited substantial protease resistance and did not display signs of cytotoxicity in cell culture even at high concentrations in the millimolar range. The perfluoroaryl cyclic and bicyclic peptides improved MF-ON analog exon-skipping activity approximately 14-fold over the unconjugated ON analogs. The bicyclic peptides exhibited increased proteolytic stability relative to the monocycle demonstrating that perfluoroaryl bicyclic arginine-rich peptides have a potential as potent and stable delivery vehicles of ON analogs. Cell culture uptake was also studied in detail by using various ON duplexes covalently labeled with Cy3-dye via internucleoside amino linkers (Figure 5C). These duplexes carried one or two MF groups (i.e. PS, 1PF-PS, 2FO-PO and 2PF-PS ON duplexes) 10. The uptake experiments demonstrated that the MF-modified ON underwent efficient internalization into A431 and INS-1 cells and that the uptake of the ON modified with MF groups was much more efficient than control ON that carried no MF groups. Mean fluorescence intensity measured in the cytoplasm of INS-1 using segmentation of fluorescence intensity images showed that INS-1 cell fluorescence was on the average 3-times higher in the case of MF-modified 2PF-PS ON duplex than PS ON duplex (Figure 6). The hybrid PO/PS ON duplexes were taken up by A431 cells much more efficiently that by INS-1 cells. The linking of additional MF residues to ON duplexes resulted in statistically significant increase of intracellular fluorescence reflecting the enhanced efficacy of uptake. ON carrying two MF groups (2PF-PS) showed considerably higher (at least 5-times) uptake in A431 cells compared to the PS control.

Several independent reports suggested that introduction of a single or multiple MF groups could help ON to escape lysosomal uptake. In particular, as indicated by LysoTracker green staining only a minimal TAMRA fluorescence signal was co-localized in the lysosomes of cells in the case of branched MF-ON 15 potentially suggesting that MF-ON molecules were either escaping from the lysosomal compartment after endocytosis, or that they were not subject to endocytosis and penetrated cells via an alternative pathway. The observed low levels of lysosomal sequestration are in agreement with the results of previous reports which indicated that fluorinated chains facilitate lysosomal escape of nanoparticles or their components 50. Since lysosomes contain various hydrolases, which cause rapid digestion of various biomolecules the observed lysosomal escape is advantageous for applications of therapeutic oligonucleotides and their analogs in gene editing and gene therapy.

### Cytotoxicity of MF-ON and their analogs

Most of the reports on MF-ON use in cell culture published so far lack systematic cytotoxicity studies. However, there is an agreement on low acute (short-term) cytotoxicity of oligonucleotides and their analogs carrying MF groups. For example, cytotoxiccity of PMO 26 and MF-ON 22 was evaluated by assessing cell viability after incubation for 5 days in Huh7 cells with increasing concentrations of MF-ON. Both reports mention the lack of cytotoxicity at high concentrations (up to 2 μM).

### The effect of MF groups on protein recognition by ON duplexes

A variety of ONs are designed so they could engage in nucleic acid-protein interaction in intracellular environment. These interactions are essential for CRISPR-cas9 gene editing, proper processing of RNAi components and for achieving transcription factor inhibition by ON duplex decoys 50-52.The effect of MF on the specificity ON duplex decoy binding to STAT3 transcription factor binding was studied by us in A431 cells 10. The duplex containing STAT3-recognition GAS sequence was labeled with near-infrared NIR Dye 800CW by using an internucleoside amino linker. As a consequence, a low background was observed facilitating high sensitivity fluorescent detection of ON duplex binding with STAT3 by using electrophoretic shift mobility assay. The assay showed that all phosphorothioate-modified ON duplexes devoid of MF groups and a control commercially available near-infrared ON probe showed similar patterns of electrophoretic mobility shift in the presence of A431 nuclear lysates. The addition of an excess of the non-labeled duplex probe resulted in the elimination of the band corresponding to a complex with STAT3, which allowed determining of the specificity of duplex-STAT3 binding. The addition of anti-STAT3 antibody to the mixture resulted in detectable supershift. The introduction of a single MF group resulted in an increase of duplex binding specificity as the intensity of a non-specific band decreased in intensity, while MF- modified duplex was still able to recognize STAT3. However, unlike non-modified duplex and the duplex carrying a single MF-group, the linking of two MF groups to opposite termini of the duplex resulted in a loss of binding to either STAT3, or to unrelated components in the lysate.

## Comparison of multifluorinated and lipid-modified oligonucleotide conjugates

There are multiple similarities between the properties of ON modified by “traditional” hydrophobic groups (such as various lipids) and MF-ON since both types of modifications have a tendency to promote the formation of supramolecular structures and to improve internalization in the cells. In both cases by introducing relatively short lipophilic or multifluorinated groups it is possible to dramatically change the properties of ON despite the fact that those groups are usually relatively small in size compared to the hydrophilic 20-25 bases-long oligonucleotide chain (or equivalent number of base pairs in the case of ON duplexes) 53. The examples of direct comparison between MF-ON and L-ON in parallel experiments are very few and while MF-modified PNA (perfluoroundecanoyl groups) showed more efficient uptake than lipid-modified PNA (2-3 time more PNA-positive cells) 24, 49, ON modified with short MF (perfluorohexyl and perfluorooctyl) groups were taken up by the cells less efficiently than L-ON 22. The authors compared intracellular uptake at 37^o^C of either 3'-fluorescein-labelled perfluorooctyl-, perfluorohexyl- modified ON, or unconjugated ON in the culture of three human cell lines (hepatic Huh7, gastric epithelial NCI-N87 and embryonic kidney HEK293). Similar to the case of hydrocarbon-modified L-ON, perfluorooctyl-ON were internalized at similar rates by all cells in all three cell lines, whereas unconjugated ONs did not penetrate into the cells under the chosen experimental conditions. Perfluorohexyl-ON was internalized less efficiently, indicating that the structure of MF group was important for the cellular internalization. To evaluate the internalization kinetics of MF-ON, Huh7 cells were incubated in the presence of 0.5 μM of MF-ON, L-ON or unconjugated ONs for either 4 or 24 h. L-ON were internalized more rapidly than both MF-ON with almost 100% of cells showed specific fluorescence after 4 h of incubation, whereas only 20% and 40% cells were fluorescent in the case of perfluorohexyl and perfluorooctyl ON, respectively. The cell uptake of both perfluorooctyl and perfluorohexyl ON was dramatically decreased at 4^o^C, which suggested that similarly to lipid-modified ON, MF-ON enter the cells via energy-dependent mechanisms (such as endocytosis or gymnosis) rather than a result of passive transition into the membrane. Unlike MF-ON, which remain at the early phase of development, L-ON were known for decades and were actively investigated for drug delivery purposes. Some of the lessons learned during the development of L-ON as pharmaceuticals may be highly relevant to the case of MF-ON. For example, it is known that L-ON interact with lipoproteins or hydrophobic surfaces resulting in substantial changes of their pharmacokinetic behavior and trans-membrane delivery 54, 55. In biological environments two competing processes, i.e. the self-assembly of L-ON into supramolecular structures (aggregation) and the ability to undergo insertion into membranes (resulting in dis-aggregation) should always be taken into account to understand and interpret their behavior 53. It is known that many highly-efficient phosphorothioate- stabilized ON may enter cells by multiple and interchangeable pathways which does not require the involvement of clathrin- and caveolin- dependent mechanisms (i.e. receptor-mediated endocytosis) 56. However, lipophilic groups appear to be re-directing L-ON toward the receptor-mediated endocytosis. The efficiency of endocytosis depends greatly on the structure of particular L-ON 53, 57. For example, double-tail lipid modification resulted in a higher degree of incorporation into both lipid model membranes and plasma membranes of the cells 57. Lipid groups linked to the termini of an ON do not result in major changes in the ability of lipid-modified ON to interact with a complementary strand since the observed change in melting of resultant duplexes does not exceed 2ºC, whereas dual modifications by using lipids located across from each other on complementary strands may result in stabilizing the resultant duplex by up to 29ºC 55, 58. Several seminal reports showed that efficient and selective uptake of L-ON (e.g. in the case of lipid-siRNA) depended on interactions with lipoprotein particles, lipoprotein receptors and transmembrane proteins 59, 60, i.e. that hydrophobicity is the primary force driving the systemic distribution of short ON duplexes such as lipid-conjugated siRNAs via lipid transport 60, 61. Recently, various modifications of siRNA were compared in terms of the effects on biodistribution after various lipid moieties (saturated and unsaturated fatty acids, steroids and vitamins) were conjugated and accumulation levels of these compounds were compared in various tissues (such as liver, spleen, and pancreas 61). The main conclusion of this study was that lipid conjugates support much broader delivery of siRNAs and enable functional silencing of target genes in many tissues, including liver, kidney, lung, heart, muscle, spleen, fat and adrenal glands. Lipid conjugate engineering can enhance extra-hepatic delivery and expand the therapeutic potential of siRNAs beyond major organs of reticuloendothelial system. An important advantage of lipid-conjugation is that its impact on biodistribution is sequence-independent 61 which broadens the potential scope of applications *in vivo*. The same appears to be true in the case of MF modifications. However, making parallel comparisons between potential effects of MF and L modifiers is not always feasible due to obvious paucity of experimental data.

## Conclusions and future perspectives

The experiments performed so far with the MF-ON (either with single ON, or ON duplexes) and their analogs carrying either one or two fluorinated “tails” show multiple drastic changes of ON behavior *in situ* and *in vitro* including the improvement of stability against degradation. Multiple reports concur on the effects of MF modifications that result in a significantly higher uptake in the cells over time *in vitro*. Compared to control nonfluorinated ON, MF-ON usually preserves biological activity. The accumulated evidence indicates the high potential of probes, RNAi components and beacons based on MF-ON for improving intracellular delivery of ONs *in vitro* and *in vivo*. Continuously evolving structural designs and improved synthon chemistry enable the development of the next generation of MF-ON, which likely will be based on ON with branched and split multifluoroalkyl tails. These designs may potentially mitigate the concerns associated with the persistence of perfluorinated acids in various environments since such newer generation of MF-ON will be a subject to biodegradation and easier excretion as some of the designs of MF-ON already suggest 62. The possible candidates could potentially include branched designs containing nonafluoro-tert-butyl group that increases fluorous effect almost as efficiently as C_8_F_17_(CH_2_)_3_ 62 or recently published C_6_F_13_-containing “split tails” (see 63 and references therein).

## Supplementary Material

Supplementary data.

## Figures and Tables

**Table 1:** Chemical structure and applications of nucleoside synthons used for synthesis of MF-ON.

Abbreviation	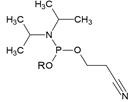 R=	Reference	Use in PFO synthesis
**Ia**	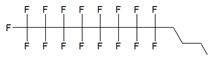	10	synthesis of 5'-modified ON
**Ib**	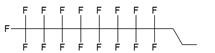	11	
**Ic**	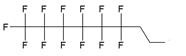	12	
**II**	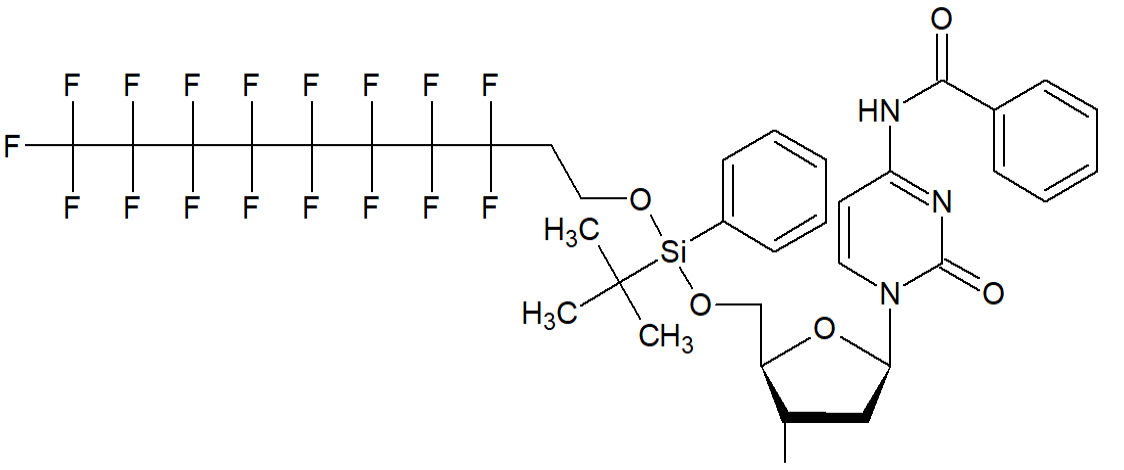	13	synthesis of 5'-modified ON
**III**	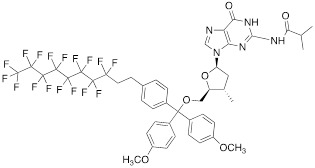	14	Fluorous affinity purification of ON
**IV**	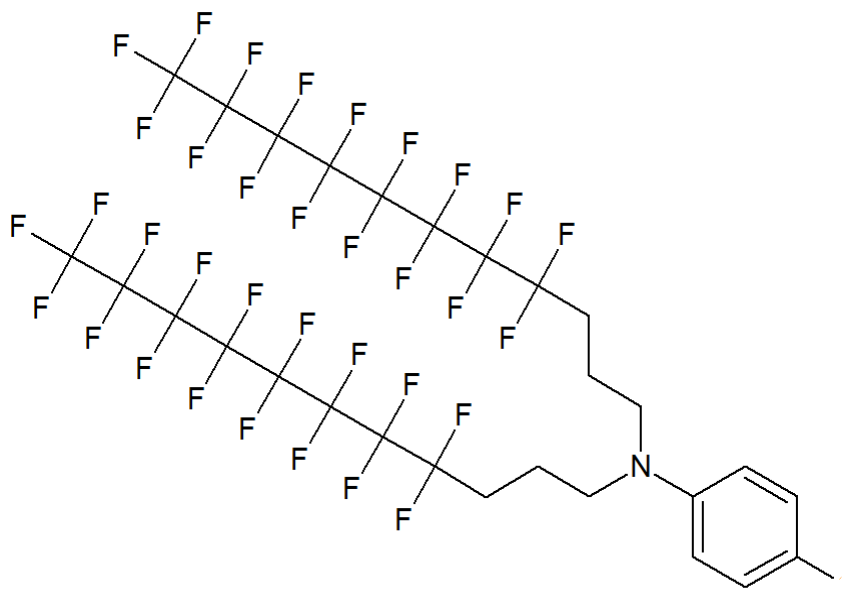	15	Synthesis of 5'-modified ON
**V**	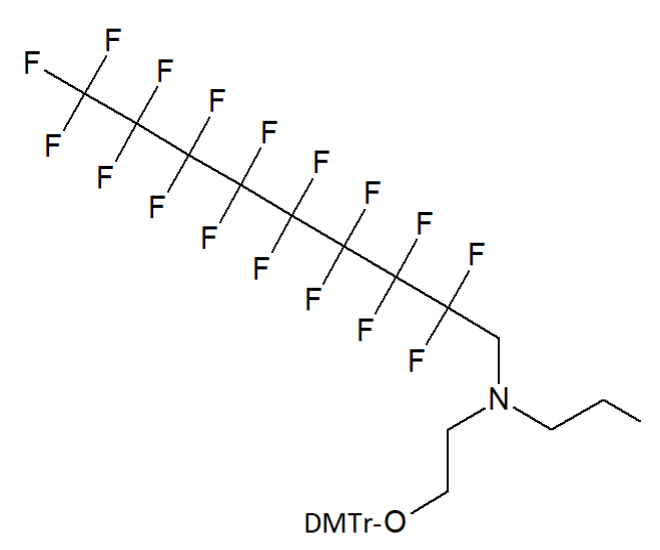	16	Introduction of C_8_F_17_-tails into ON
**VI**	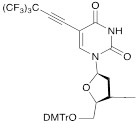	17	Introduction of 5-MF-2'-deoxyuridine into any position of ON chain
**VIIa**	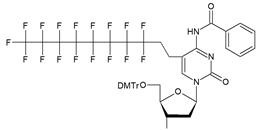 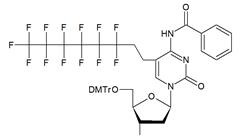	18	Introduction of 5-MF-2'-deoxyuridine into any position of ON chain
**VIIb**			
**VIIIa**	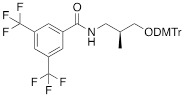	19	Introduction of “**F**-bases” into any position of ON chain
**VIIIb**	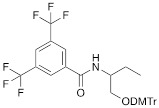	20	Synthesis of ON containing 3,5-bis(trifluoromethyl) benzene
**VIIIc**	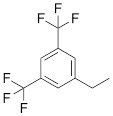	21	ON with 3,5-bis (trifluoromethyl) benzene in 5'-position
**IX**	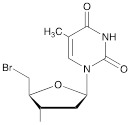	22	5'-bromo-5'-deoxythymidine on 5'- end of ON for subsequent synthesis of azido derivatives
**X**	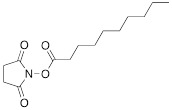	10	ON with activated group on 5'-end for post synthetic modification with perfluorinated amines
**XI**	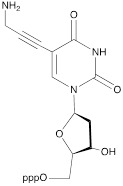	23	Enzymatic primer extension synthesis of ONs

**Table 2:**
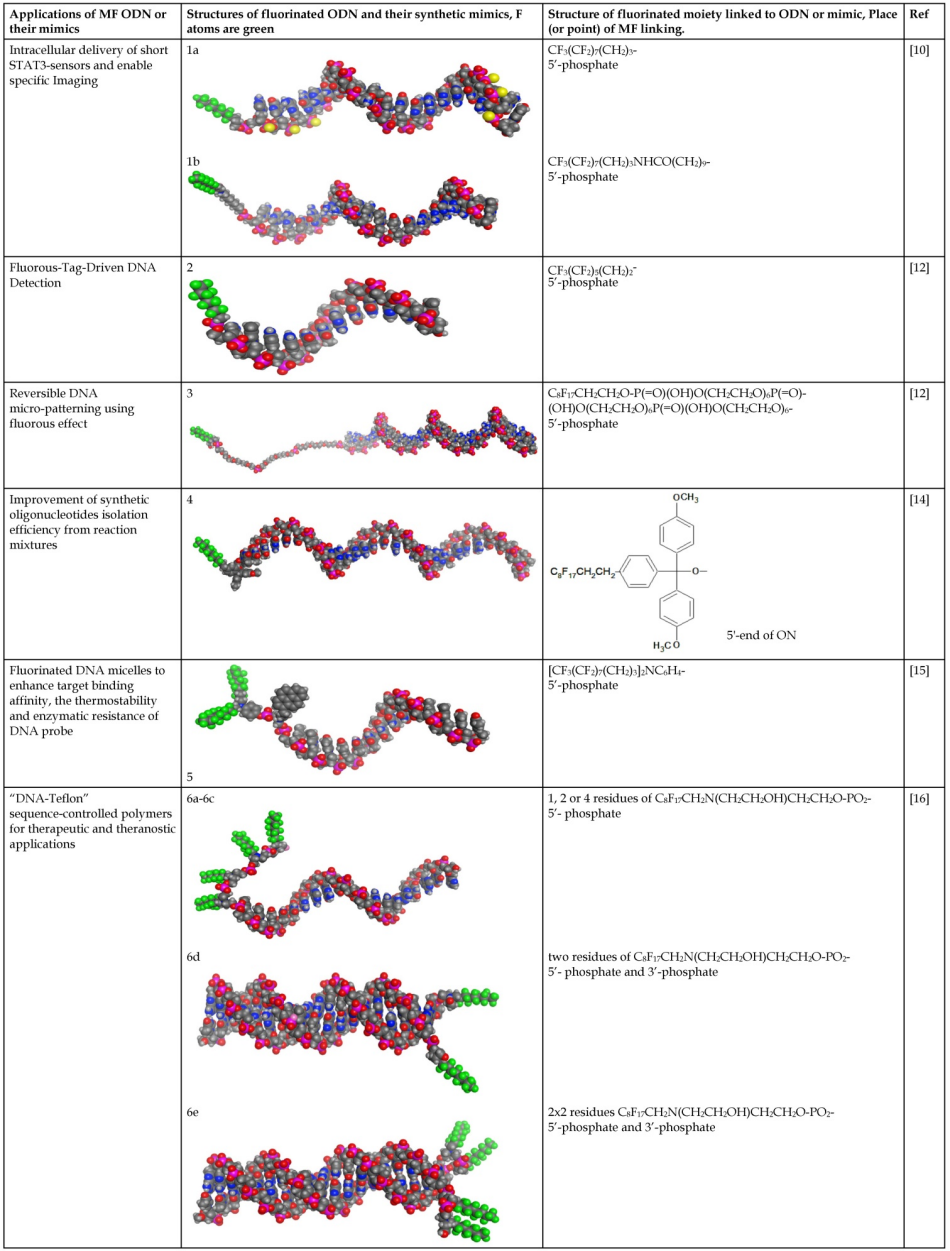

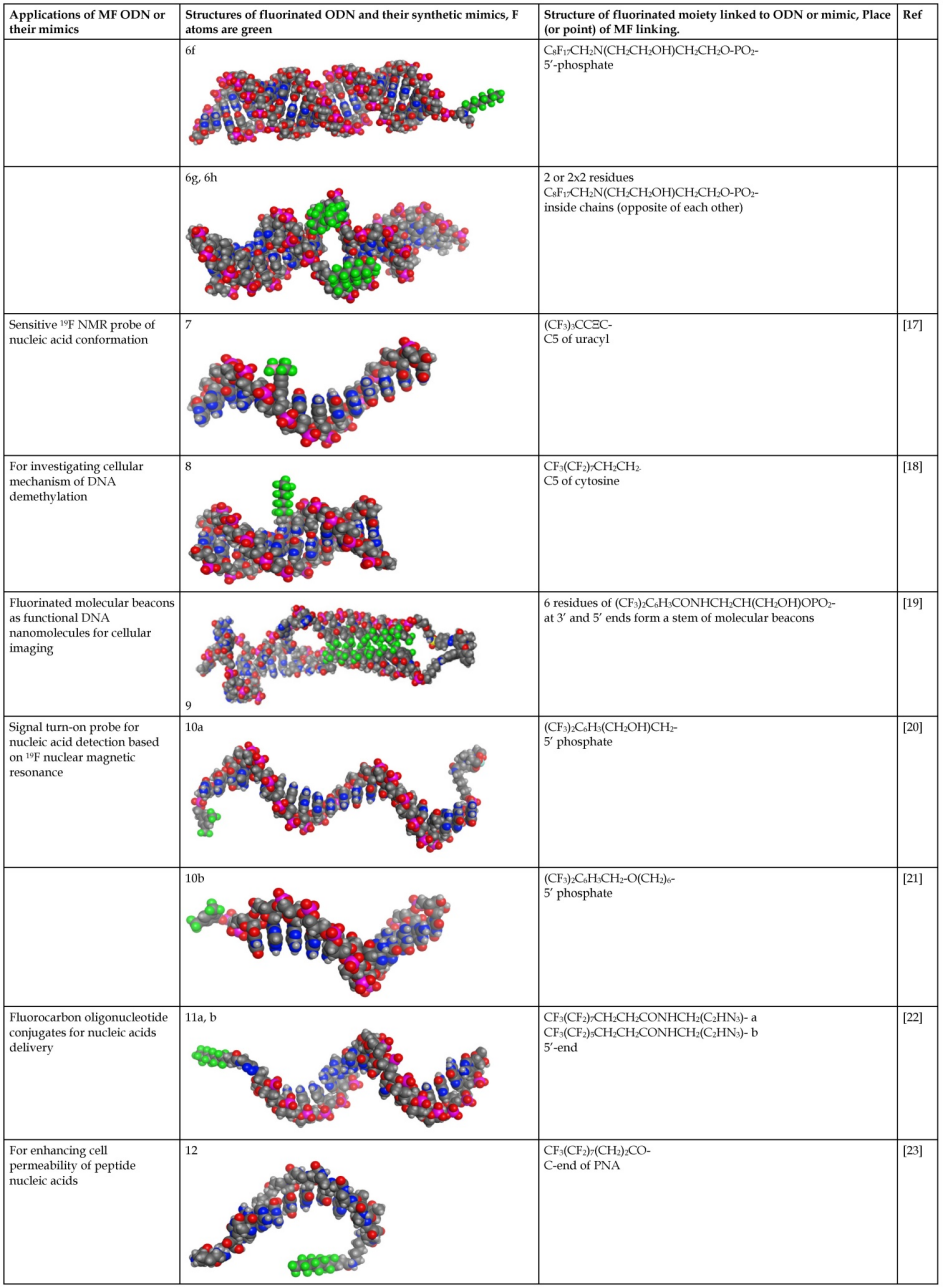

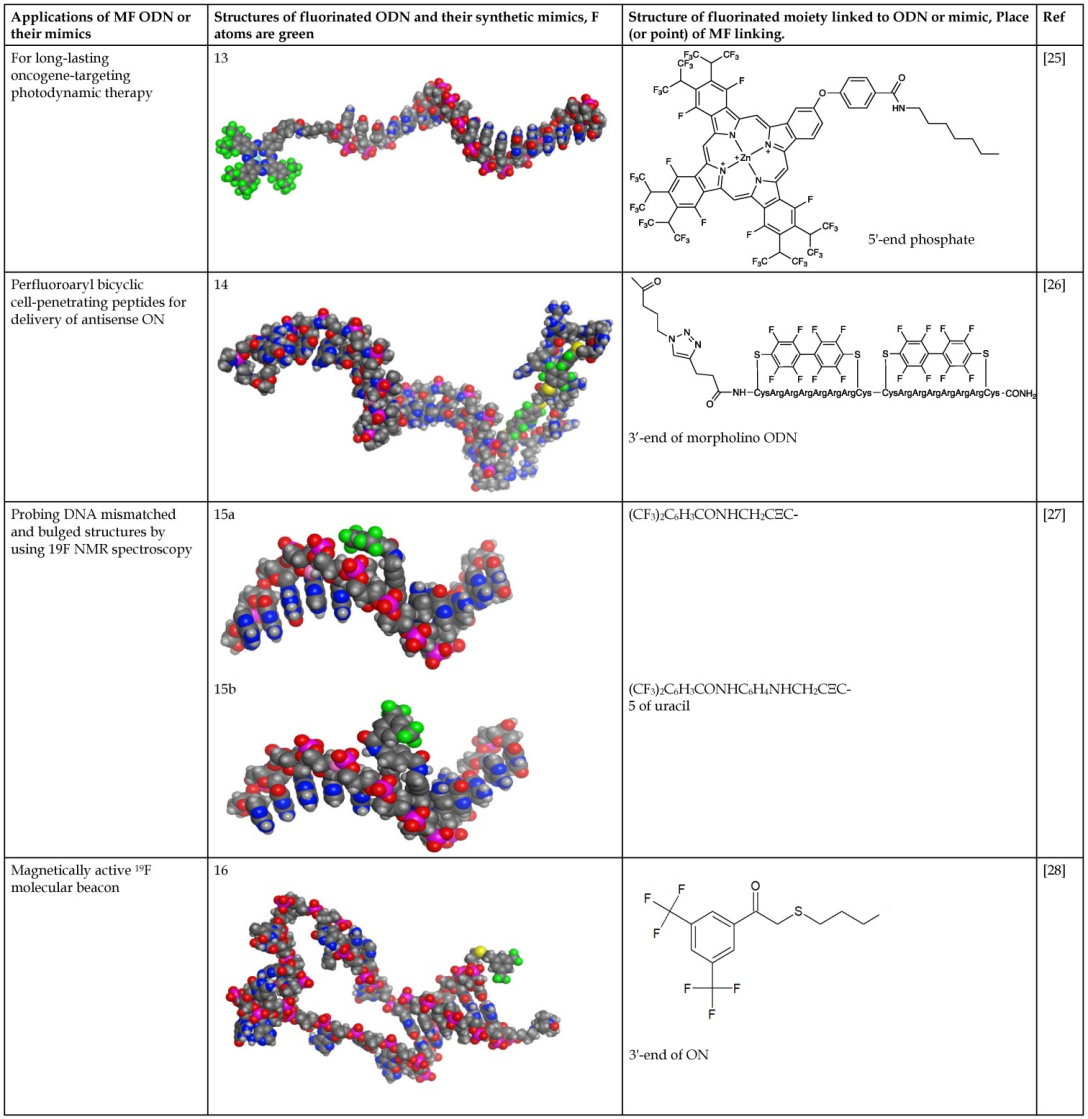
Structure and use of fluorinated oligonucleotides.

Molecular models of fluorinated ODN and their synthetic mimics were performed by using MOE2016 and MOE2017 software (Chemical Computing Group, Montreal CA) 29. Fluorine atoms are color coded in green. Data is available in pdb format (see Supplemental information).

## Abbreviations

DMTr: 4,4'-dimethoxytrityl
F base: artificial nucleotides with 3,5-bis(trifluoromethyl)benzene moieties
FCN: 9-[3-(perfluorooctyl)propylcarbamoyl]nonyl
FO: CF_3_(CF_2_)_7_(CH_2_)_3_-
GAS: gamma interferon activation site
HBTU: 3-[bis(dimethylamino)methyliumyl]-3*H*-benzotriazol-1-oxide hexafluorophosphate
HOBt: benzotriazol-1-ol
L-ON: lipid conjugated oligonucleotide
MF: multifluorinated
MF-ON: multi-fluorinated oligodeoxynucleotide
ON: oligodeoxynucleotide
PMO: phosphorodiamidate morpholino oligonucleotide
PNA: peptide nucleic acid
STAT3: signal transducer and activator of transcription 3
TAMRA: 5-carboxytetramethylrhodamine
